# NDUFS4 Regulates Cristae Remodeling in Diabetic Kidney Disease

**DOI:** 10.21203/rs.3.rs-3070079/v1

**Published:** 2023-06-30

**Authors:** Koki Mise, Jianyin Long, Daniel L. Galvan, Zengchun Ye, Guizhen Fan, Irina I. Serysheva, Travis I. Moore, Jun Wada, Paul T. Schumacker, Benny H. Chang, Farhad R. Danesh

**Affiliations:** 1 Section of Nephrology, The University of Texas MD Anderson Cancer Center, Houston, Texas; 2 Department of Nephrology, Rheumatology, Endocrinology & Metabolism, Okayama University Graduate School of Medicine, Dentistry & Pharmaceutical Sciences, Okayama, Japan; 3 Department of Hematopathology, The University of Texas MD Anderson Cancer Center, Houston, Texas; 4 Division of Nephrology, The Third Affiliated Hospital of Sun Yat-Sen University, Guangzhou, China; 5 Department of Biochemistry and Molecular Biology, The University of Texas Health Science Center at Houston, Houston, Texas; 6 Department of Integrative Biology and Pharmacology, The University of Texas Health Science Center at Houston, Houston, Texas; 7 Department of Pediatrics, Feinberg School of Medicine, Northwestern University, Chicago, IL; 8 Department of Pharmacology and Chemical Biology, Baylor College of Medicine, Houston, TX

## Abstract

The mitochondrial electron transport chain (ETC) is a highly adaptive process to meet metabolic demands of the cell, and its dysregulation has been associated with diverse clinical pathologies. However, the role and nature of impaired ETC in kidney diseases remains poorly understood. Here, we generated diabetic mice with podocyte-specific overexpression of Ndufs4, an accessory subunit of mitochondrial complex I, as a model to investigate the role of ETC integrity in diabetic kidney disease (DKD). We find that these conditional mice exhibit significant improvements in cristae morphology, mitochondrial dynamics, and albuminuria. By coupling proximity labeling with super-resolution imaging, we also identify the role of cristae shaping proteins in linking NDUFS4 with improved cristae morphology. Taken together, we discover the central role of NDUFS4 as a powerful regulator of cristae remodeling, respiratory supercomplexes assembly, and mitochondrial ultrastructure *in vitro* and *in vivo*. We propose that targeting NDUFS4 represents a promising approach to slow the progression of DKD.

Diabetic kidney disease (DKD), a serious microvascular complication of diabetes, is the leading cause of end-stage kidney disease worldwide^1^. Recent studies have shown promising results with the use of sodium-glucose cotransporter-2 (SGLT2) inhibitors and glucagon-like peptide-1 (GLP-1) receptor agonists in reducing the risk of cardiovascular disease and progression of DKD^2,3^. However, despite recent advances, the risk of progression to renal replacement therapy and kidney transplantation in individuals with DKD remains high, underscoring the need for continued research into the underlying molecular mechanisms involved in the pathobiology of DKD and the development of novel therapies to address this unmet need.

Among multiple molecular mechanisms that have been implicated in the pathogenesis of DKD^4^, the role of mitochondrial dysfunction in DKD has become increasingly recognized^5–7^. Indeed, several recent studies have identified a spectrum of morphologic and biochemical abnormalities of mitochondria in the diabetic environment, including the production of reactive oxygen species (ROS), changes in mitochondrial biogenesis, and significant remodeling of mitochondrial dynamics^8–10^. However, despite considerable progress in understanding the role of mitochondrial remodeling in kidney cells^11,12^, the exact role and nature of mitochondrial dysfunction in the diabetic environment is incompletely understood.

The “unifying theory of microvascular complications of diabetes”, a concept conceived by Brownlee and colleagues, proposed that mitochondrial dysfunction in microvascular complications of diabetes could be defined as an excess production of mitochondrial ROS (mROS) through impaired ETC function^13,14^. However, the conclusions drawn from this study were widely challenged because of the lack of *in vivo* evidence to link mitochondrial respiration defects with mROS generation and the pathogenesis of DKD.

Maintenance of mitochondrial respiration requires the proper assembly and function of four mitochondrial enzymes (complex I–IV; CI–IV), collectively known as electron transport chain (ETC), embedded within the inner membrane of the mitochondrion to allow for the transfer of electrons from the substrates to oxygen and generation of an electrochemical gradient^15^. Mitochondrial complex I (CI, NADH: ubiquinone oxidoreductase), the first and the largest component of ETC (~1 MDa), is classically considered the entry point for electrons into the ETC that couples the transfer of two electrons from NADH to ubiquinone with the translocation of four protons across the inner mitochondrial membrane^16^. CI also plays an essential role in biosynthesis and redox activities of the mitochondria^17,18^. In mammals, CI is a highly organized L-shaped holoenzyme that is comprised of 14 conserved catalytic “core subunits” and 31 “accessory or supernumerary subunits” ^19^. Recent evidence suggests that CI can exist independently or in the form of multi-complex assemblies at a higher level of organization, consisting of CI, III and IV together in different stochiometric ratios, known as respiratory supercomplexes (RSCs) within the inner membrane of the cristae^20^. RSC formation has been proposed to enhance stability of individual complexes in addition to preventing electrons from escaping, and therefore limiting the formation of mROS^21,22^.

ETC dysfunction is recognized as an important cause of organ failure in several human pathologies including heart failure, diabetes, and neurodegeneration in a tissue-specific manner^23–26^. In the current study, we reasoned that ETC remodeling in kidney podocytes might contribute to mitochondrial dysfunction in the diabetic environment with the subsequent progression of DKD. To this end, we set out to test the interplay between DKD progression and ETC remodeling in the kidney podocytes and whether these changes correlate with the aberrant mitochondrial morphology in the diabetic milieu. We examined ETC remodeling in podocytes in the diabetic environment and generated mice with podocyte-specific overexpression of Ndufs4 (NADH: ubiquinone oxidoreductase iron-sulfur protein 4), an accessory subunit of CI, as a model to investigate the role of ETC integrity in DKD progression. Our results unexpectedly reveal that ETC integrity determines the stability of RSCs and plays a central role in cristae and mitochondrial dynamics in podocytes.

## RESULTS

### ETC remodeling of podocytes in DKD.

**Extended Data Fig. 1 |:**
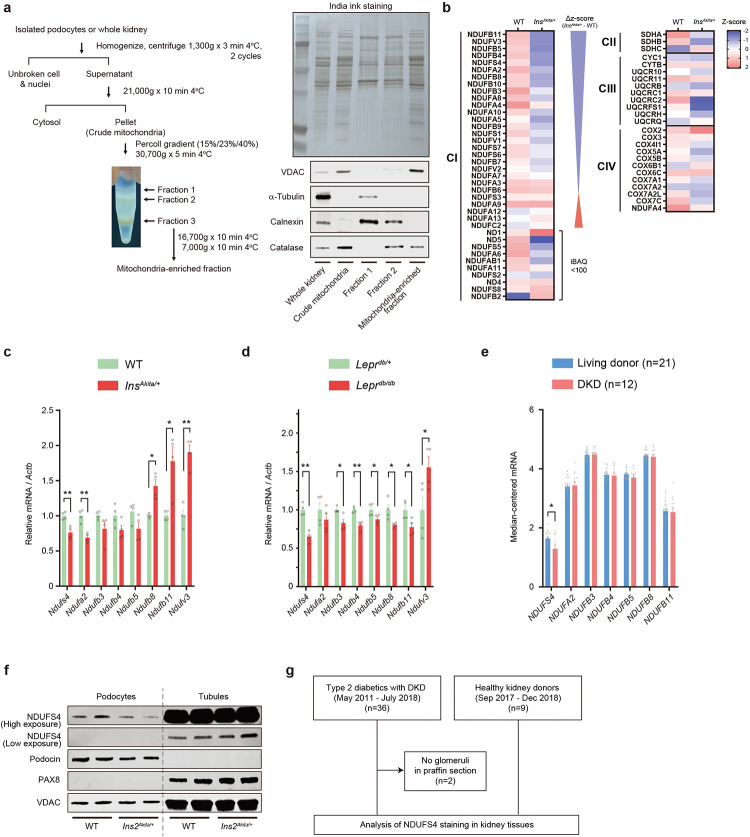
NDUFS4 expression is downregulated in podocytes, but not in tubules, in diabetic environment. **a**, Mitochondria isolation from podocytes or whole kidney using Percoll density gradient centrifugation (left). Immunoblots to validate the purity of mitochondria in different fractions (lower right). India ink staining was used to show all proteins (upper right). **b**, Proteomic profiling of mitochondrial proteins from primary podocytes of WT and *Ins2*^*Akita/+*^ mice. Heatmaps of CI subunits (left panel), and CII-CIV subunits (right panel). **c,d**, qRT-PCR analysis of the relative mRNA expression of selective CI subunits (relative to *Actb*) in isolated podocytes from 16-week-old WT/ *Ins2*^*Akita/+*^ (**c**) and *Lepr*^*db/+*^/*Lepr*^*db/db*^ mice (**d**) (n=4, each n represents a pool of RNA samples from 4 different mice). **e**, mRNA expression of selective CI subunits in glomeruli of human DKD (Nephroseq v5) (Healthy living donor n=21, DKD patients n=12), Median-centered Log_2_ values are used for the analysis. **f**, Western blot analysis of NDUFS4 in primary podocytes and tubular cells isolated from WT and *Ins2*^*Akita/+*^ mice, Podocin (podocyte marker), PAX8 (tubular marker), and VDAC (mitochondrial marker) were used as cell type specific markers. Exp: exposure. **g**, Study flowchart of the NDUFS4 staining analysis in kidney biopsies from DKD patients (n=34) and healthy donors (n=9). Data are mean ± SEM. *P < 0.05, **P < 0.01, unpaired two-sided *t* test, FDR *Q*< 0.05 (**c**-**e**).

**Fig. 1 |:**
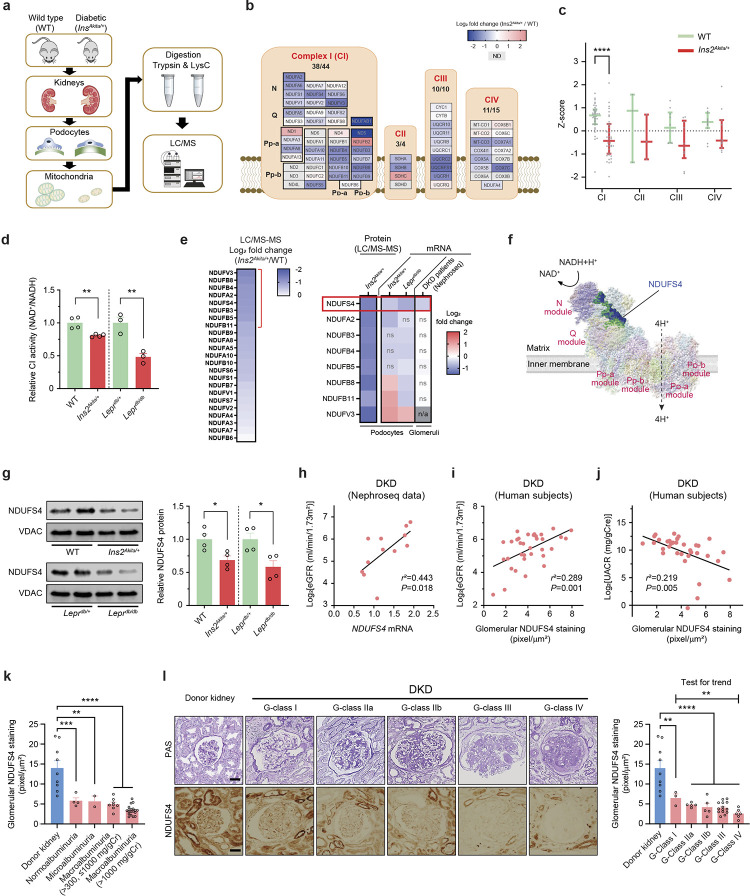
Reduced Ndufs4 expression in podocytes in DKD. **a**, Experimental workflow of quantification and comparison of mitoproteomes in primary mouse podocytes from diabetic *Ins2*^*Akita/+*^ vs. WT mice (n=8/group). **b**, Heatmap illustrating log_2_ fold change in mitochondrial ETC protein abundance in diabetic *Ins2*^*Akita/+*^ *vs.* WT mice. **c**, Aggregated abundance of ETC proteins in podocytes of WT *vs.* diabetic *Ins2*^*Akita/+*^ mice. Data are presented as median ± inter quartile range (IQR). **d**, CI activity in mitochondrial enriched samples isolated from primary podocytes (n=3–4 where each sample represents a pool of mitochondrial enriched samples from 4–6 mice). **e**, qRT-PCR validation of selected reduced CI subunits from Fig. 1b. Expression levels of mRNA subunits in diabetic subjects with DKD was obtained from Nephroseq v5 dataset (nephroseq.org; Ju CKD glom database). ns: not significant and n/a: not available. **f**, Human CI structure highlighting the location of NDUFS4 (blue) in the N module of the matrix arm of CI. The structure visualization was rendered through UCSF ChimeraX software (www.rbvi.ucsf.edu/chimerax). **g**, Left panel, representative immunoblots of NDUFS4 using mitochondria-enriched samples from primary podocytes of WT, *Ins2*^*Akita/+*^, *Lepr*^*db/+*^, and *Lepr*^*db/db*^ mice. VDAC was used as a loading control. Right panel, densitometric analysis of western blots normalized to control levels (n=4, each n represents a pool of mitochondria from 4 different mice). **h**, Pearson’s correlation analysis of human *NDUFS4* mRNA expression. Data obtained from Nephroseq v5 (n=12). Median-centered Log_2_ values are used for the analysis. **i**,**j**, Pearson’s correlation of glomerular NDUFS4 immunostaining with eGFR (**i**) and UACR (**j**) from diabetic subjects with DKD (n=34). **k**, Representative NDUFS4 immunostaining in glomeruli obtained from biopsies from healthy donors (n=9) and DKD patients with various degrees of albuminuria, including DKD with normoalbuminuria (n=4), DKD with microalbuminuria (n=2), DKD with macroalbuminuria (UACR >300, ≤1000 mg/gCr, n=8), and DKD with macroalbuminuria (UACR >1000mg/gCr, n=19). **l**, Glomerular NDUFS4 immunostaining in biopsies from healthy donors and DKD individuals with different stages of glomerular involvement: Donors (n=9), Glomerular (G)-class I DKD (n=3), G-class IIa DKD (n=5), G-class IIb DKD (n=5), G-class III DKD (n=14), G-class IV DKD (n=6), Scale bars= 50 μm. Data are presented as mean ± SEM except for (**c**). *P< 0.05, **P < 0.01, ***P < 0.001, and ****P < 0.0001 by Mann-Whitney test, FDR *Q*< 0.01 (**c**), unpaired two-sided *t* test (**d**,**g**), one-way ANOVA with post-hoc Tukey’s test (**k**,**l**), test for trend analysis for different classifications of DKD (**l**)

ETC is known to exhibit significant remodeling in response to the metabolic demands of the cell^16^. However, it remains unclear how ETC adapts to the diabetic milieu in podocytes. To provide a deeper insight into the possible dynamics of individual ETC complexes in the diabetic environment, we performed a comparative mitochondrial proteome profiling focusing quantitatively on the protein abundance of ETC complexes in primary podocytes isolated from diabetic C57BL/6-*Ins2*^*Akita*^/J *(Ins2*^*Akita/+*^) mice, an established model of type 1 diabetes, and their nondiabetic littermates (Fig. 1a and Extended Data Fig. 1a). We identified 62 out of the 73 known subunits of mouse ETCs corresponding to a recovery rate of 85% (Fig. 1b). Notably, we found that the abundance of several subunits of CI was significantly reduced in the podocytes of diabetic mice (Fig. 1b,c and Extended Data Fig. 1b). Consistent with this finding, we also observed reduced CI enzymatic activity in enriched mitochondrial samples from podocytes in both type 1 (*Ins2*^*Akita/+*^) and type 2 (*Lepr*^*db/db*^) diabetic mice (Fig. 1d). Among the most prominently reduced CI subunits validated in a series of quantitative RT-PCR experiments (Fig. 1e and Extended Data Fig. 1c–e), we focused on Ndufs4, an accessory 18 kDa subunit of the CI, for further analysis since its mRNA and protein levels were consistently reduced not only in podocytes of type 1 (*Ins2*^*Akita/+*^) and type 2 (*Lepr*^*db/db*^) diabetic mouse models but also in glomeruli of subjects with DKD (Fig. 1e–g and Extended Data Fig. 1c–e). Interestingly, NDUFS4 protein expression in the kidney tubular cells remains unchanged in diabetic mice (Extended Data Fig. 1f). Using Nephroseq database, we found a positive correlation between *NDUFS4* mRNA in glomeruli and estimated glomerular filtration rate (eGFR) in subjects with DKD (Fig. 1h). We validated these findings by immunohistochemistry (IHC) on paraffin sections in a cohort of 34 subjects with established DKD (Extended Data Fig. 1g and Supplementary Table 1). We found that the glomerular NDUFS4 expression correlated positively with eGFR (*r*^2^=0.289, *P*=0.001; Fig. 1i) and negatively with the urinary albumin excretion rate (UACR) (*r*^2^=0.219, *P*=0.005; Fig. 1j). Importantly, NDUFS4 staining was significantly decreased in the kidney glomeruli of diabetic subjects with normoalbuminuria as compared to that of healthy donors (5.8 ± 0.7 *vs.* 14.0 ± 1.9 pixel/μm^2^, *P*<0.001; Fig. 1k), suggesting that NDUFS4 downregulation may have occurred prior to the clinical manifestation of DKD. We also evaluated NDUFS4 staining in glomeruli from diabetic subjects with a wide spectrum of DKD histology^27^, and found that NDUFS4 staining in glomeruli was progressively reduced with worsening of DKD histology (test for trend *P*<0.01) (Fig. 1l). Taken together, the consistently reduced expression of Ndufs4 in both murine models of diabetes and in human DKD subjects suggest that Ndufs4 may play an important role in progression of DKD.

### Podocyte-specific overexpression of Ndufs4 mitigates the progression of DKD.

**Extended Data Fig. 2 |:**
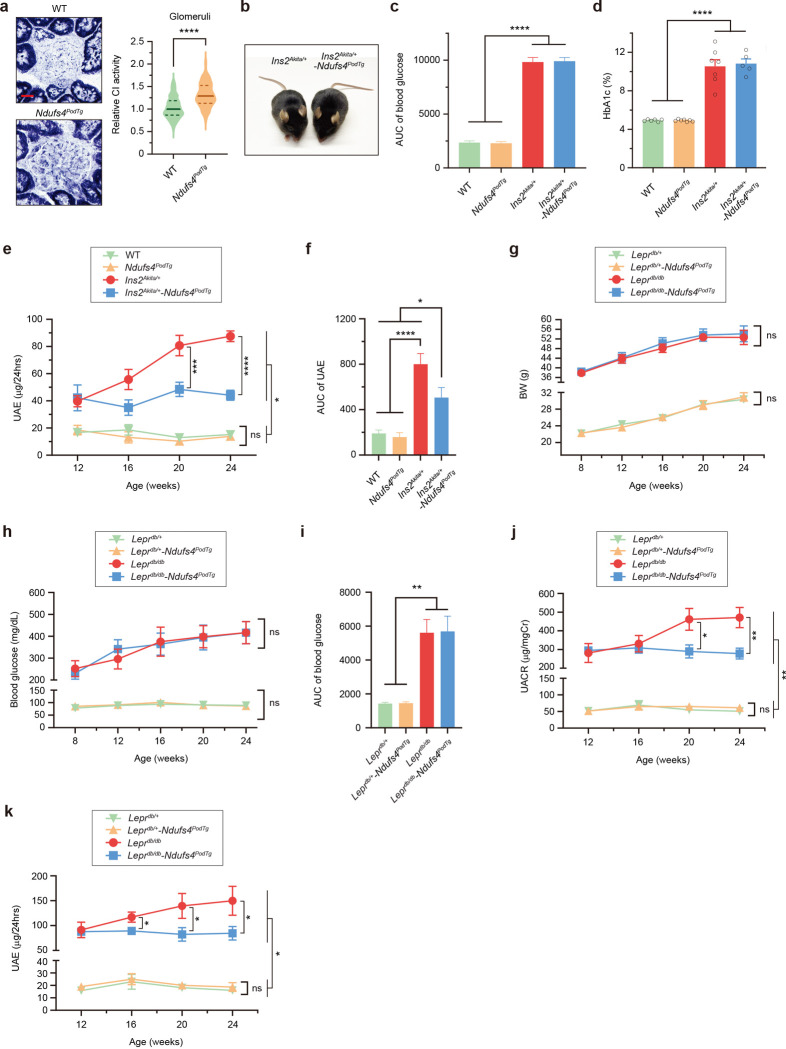
Podocyte-specific Ndufs4 overexpression mitigates albuminuria in type 2 diabetic mice. **a**, Representative images of NADH oxidoreductase (CI) activity in glomeruli from WT and *Ndufs4*^*PodTg*^ mice (left panel). Relative CI activity in glomeruli (right panel) (n=150 from 3 mice/group). Scale bar=20 μm. **b**, Appearance of a diabetic (*Ins2*^*Akita/+*^) mouse and a diabetic *Ndufs4*^*PodTg*^ mice at 18 weeks of age. **c**, Area under the curve (AUC) of blood glucose levels from 4-, 10-, 14-, 18- and 23-week-old mice shown in Fig. 2f. **d**, Hemoglobin A1C (HbA1c) levels in four different groups of mice. **e**, Urinary albumin excretion (UAE, μg/24hrs) in 12-, 16-, 20- and 24-week-old mice. **f**, AUC of UAE shown in Extended Data Fig. 2e. **g**-**k**, Body weight (**g**), blood glucose (**h**), blood glucose AUC (**i**), urinary albumin-to-creatinine ratio (UACR) (**j**), and UAE (**k**) of control and type 2 diabetic *Lepr*^*db/db*^ mice at different ages (n=7–9/group). Results are presented as median ± IQR (**a**, bold line: median, and dot line: IQR) or mean ± SEM (**c**-**k**). *P < 0.05, ** *P* < 0.01, *** *P* < 0.001, **** *P* < 0.0001. Mann-Whitney test (**a**), One-way ANOVA with post-hoc Tukey’s test (**c**-**k**).

**Fig. 2 |:**
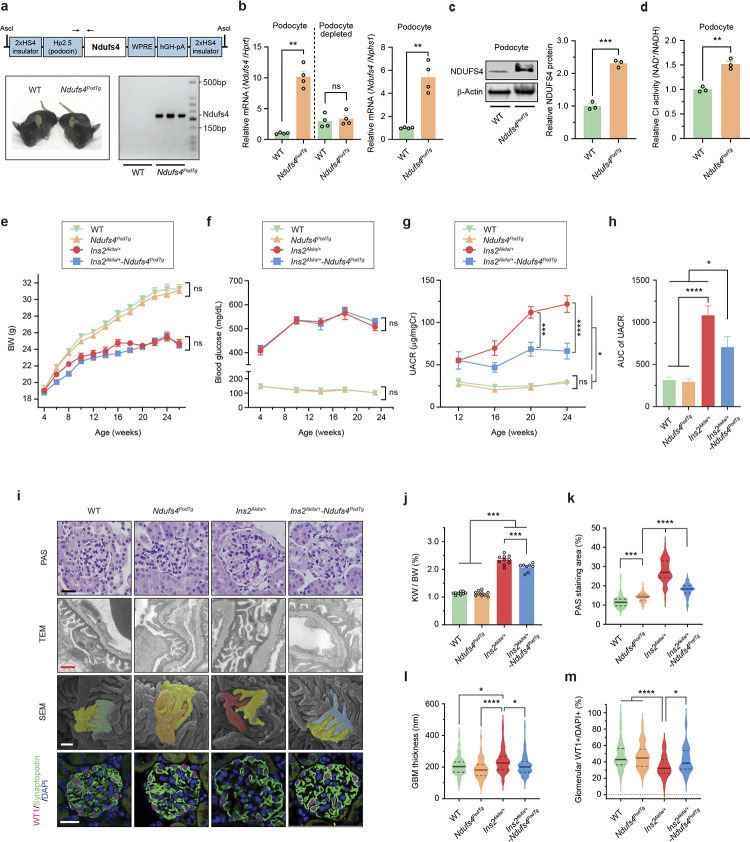
Podocyte-specific Ndufs4 overexpression ameliorates DKD progression. **a**, Schematic depiction of the construct used to engineer podocyte-specific Ndufs4-transgenic mice (*Ndufs4*^*PodTg*^) (top), representative images of WT and transgenic mice (lower left), and PCR genotyping (lower right). **b**, qRT-PCR of *Ndufs4* mRNA in primary podocytes and podocytes-depleted samples isolated from 8-week-old WT and *Ndufs4*^*PodTg*^ mice. (n=4). **c**, A representative NDUFS4 immunoblot (left), and quantification of NDUFS4 protein expression (right) (n=3). **d**, CI enzymatic activity in podocytes isolated from 8-week-old WT and *Ndufs4*^*PodTg*^ mice (n=3). **e**-**g**, Body weight (BW) (**e**), blood glucose (**f**), and urinary albumin-to-creatinine ratio (UACR) (**g**) in mice (n=8–12/group). **h**, Area under the curve (AUC) of UACR shown in Figure 2G (n=8–12/group). **i**, Representative micrographs of Periodic acid-Schiff (PAS) stained kidney sections, podocyte morphology in transmission electron microscopy (TEM), scanning electron microscopy (SEM), and immunostaining of Wilms’ tumor 1 (WT1) (pink), Synaptopodin (green), and DAPI (blue). Scale bars=50 μm in row 1, 500 nm in rows 2 and 3, and 50 μm in row 4. **j**, Kidney weight (KW) per body weight (BW) of 26-week-old mice (n=8–10/group). **k**, PAS positive area in glomeruli (n=60 from 3 mice/group). **l**, Glomerular basement membrane thickness (n=99–105 areas of TEM images from 3 mice/group). **m**, WT1 positive nuclei in glomeruli (n=60 from 3 mice/group). Data are presented as mean ± SEM (**b**-**h,j**) or median ± IQR (**k**-**m**, bold line: median, and dot line: IQR). ns, not significant; *P < 0.05, **P < 0.01, ***P < 0.001, ****P < 0.0001; unpaired two-sided Welch’s *t* test (**b**-**d**), one-way ANOVA with post-hoc Tukey’s test (**e-h,j**), or Kruskal-Wallis with post-hoc Dunn’s test (**k**-**m**).

To assess the potential role of reduced NDUFS4 in the development of DKD, we engineered a podocyte-specific Ndufs4 transgenic mouse model (*Ndufs4*^*podTg*^) (Fig. 2a). Hemizygous *Ndufs4*^*podTg*^ mice were indistinguishable from wild-type (WT) mice (Fig. 2a). Compared to podocytes isolated from age-matched littermate WT mice, there was ~2-fold increase in protein expression and ~5-fold higher mRNA expression of Ndufs4 in primary podocytes of *Ndufs4*^*podTg*^ mice (Fig. 2b,c). Notably, podocytes from *Ndufs4*^*podTg*^ mice exhibited a higher NADH oxidoreductase (CI) activity by ~50% whereas CI activity in *Ndufs4*^*podTg*^ glomeruli was ~30% higher than WT controls (Fig. 2d and Extended Data Fig. 2a). We next crossed *Ndufs4*^*podTg*^ mice with diabetic *Ins2*^*Akita/+*^ mice to generate diabetic *Ins2*^*Akita/+*^*;Ndufs4*^*podTg*^ (herein diabetic *Ndufs4*^*podTg*^) mice. Diabetic *Ndufs4*^*podTg*^ mice did not differ in body weight, blood glucose, and HbA1c when compared to diabetic *Ins2*^*Akita/+*^ mice (Fig. 2e,f and Extended Data Fig 2b–d). However, they exhibited significant protection against biochemical and histological features of DKD, including albuminuria (Fig. 2g,h and Extended Data Fig 2e,f), kidney and glomerular hypertrophy (Fig. 2i,j), mesangial matrix expansion (Fig. 2k), glomerular basement membrane (GBM) thickening (Fig. 2l), podocyte foot process effacement and podocytes loss (Fig. 2i,m). We also crossed *Ndufs4*^*podTg*^ mice with obese *Lepr*^*db/db*^ mice, an established model of type 2 diabetes, and found similar phenotypes with a significant improvement in albuminuria in diabetic *Lepr*^*db/db*^*;Ndufs4*^*podTg*^ independent of body weight gain and blood glucose levels (Extended Data Fig. 2g–k).

### Ndufs4 overexpression improves mitochondrial morphology.

**Fig. 3 |:**
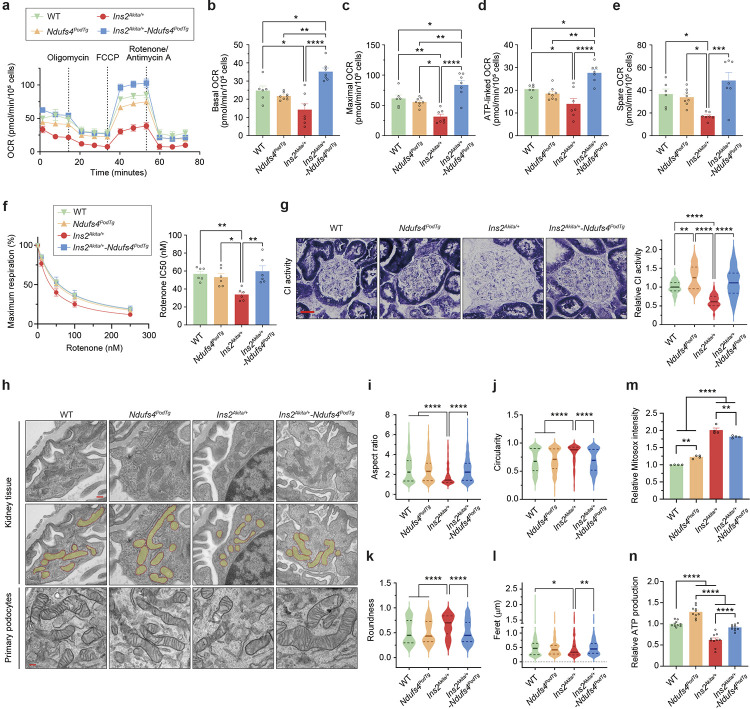
Ndufs4 overexpression improves mitochondrial morphology in the diabetic environment. **a**, OCRs in primary podocytes (n=6–8 replicates/group). Dotted lines denote injections of oligomycin (2 μM), FCCP (2 μM), rotenone, and antimycin A (both 0.5 μM). **b**-**e**, Basal respiration (**b**), maximal respiration (**c**), ATP-linked OCR (**d**), and spare OCR (**e**). **f**, Primary podocytes susceptibility to rotenone (left panel), and rotenone half maximal inhibition (IC_50_) (right panel, n=6 replicates/group). **g**, Representative glomerular CI activity in different group of mice (n=60 from 3 mice/group, scale bar=50 μm). **h**, Representative podocyte mitochondria from kidney tissues (top row) and pseudo-color superimposed images (middle row) to assess mitochondrial morphological changes, including aspect ratio (**i**), circularity (**j**), roundness (**k**), and feret diameter (**l**) (n=141–312 from 3 mice/group). TEM micrographs from primary podocytes isolated from experimental mice (bottom row). Scale bars=200 nm. **m**,**n**, Mitochondrial ROS assessed by Mitosox Red staining (n=4 replicates/group) (**m**), and relative ATP production (n=10 replicates/group) (**n**). Data are presented as mean ± SEM (**a**-**f**,**m**,**n**) or median ± IQR (**g**,**i**-**l**, bold line: median, and dot line: IQR). *P < 0.05, **P < 0.01, ***P < 0.001, ****P < 0.0001. One-way ANOVA with post-hoc Tukey’s test (**b**-**f**,**m**,**n**), or Kruskal-Wallis with post-hoc Dunn’s test (**g**,**i**-**l**).

We reasoned that the underlying molecular mechanism of *Ndufs4*^*podTg*^-mediated improvement in DKD could be associated with improved mitochondrial respiration in podocytes. To test this, we measured mitochondrial respiration using a Seahorse Analyzer. Whereas the oxygen-consumption-rate (OCR) measurements were similar between primary podocytes from WT and *Ndufs4*^*podTg*^ mice, podocytes from diabetic *Ins2*^*Akita/+*^ mice exhibited a significantly reduced basal, maximal, ATP-linked, and spare OCR values (Fig. 3a–e). In contrast, OCR values were markedly improved in podocytes from diabetic *Ndufs4*^*podTg*^ mice (Fig. 3a–e). We next tested the susceptibility of these podocytes to rotenone, a CI-specific inhibitor. We found that although podocytes from diabetic *Ins2*^*Akita/+*^ mice had a significantly lower OCR suppression curve and rotenone IC_50_ values, primary podocytes from diabetic *Ndufs4*^*podTg*^ mice had much higher IC_50_ and OCR suppression curve, almost similar to those in WT mice (Fig. 3f). Consistent with these findings, we also found improved CI activity in the glomeruli of the diabetic *Ndufs4*^*podTg*^ mice compared to that in podocytes from *Ins2*^*Akita/+*^ mice (Fig. 3g).

**Extended Data Fig. 3 |:**
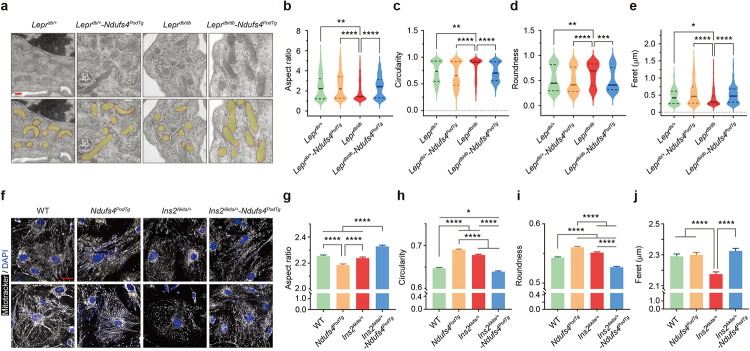
Ndufs4 overexpression prevents mitochondrial fission in podocytes from type1 and type 2 diabetic mice. **a**-**e**, Representative TEM micrographs of podocyte mitochondria from kidney tissues (**a**, top) and pseudo-color superimposed images (**a**, bottom) to assess mitochondrial morphological changes in aspect ratio (**b**), circularity (**c**), roundness (**d**), and feret diameter (**e**) (n=115–454 from 3 mice/group, Scale bar=200 nm). **f**-**j**, Representative immunofluorescent images (**f**) of primary podocytes stained with mitotracker (white) showing mitochondrial morphological changes in aspect ratio (**g**), circularity (**h**), roundness (**i**), and feret diameter (**j**) (n=21675–23513/group, Scale bar=40 μm). Data are presented as median ± IQR (**b**-**e**, bold line: median, and dot line: IQR) or mean ± SEM (**g**-**j**). *P < 0.05, **P < 0.01, ***P < 0.001, ****P < 0.0001. Kruskal-Wallis with post-hoc Dunn’s test (**b**-**e**) and One-way ANOVA with post-hoc Tukey’s test (**g**-**j**).

We have previously shown that DKD progression is associated with an excessive mitochondrial fission in podocytes^28,29^. Thus, we next explored the contributions of *Ndufs4*^*podTg*^ on mitochondrial dynamics. We confirmed that podocytes from type 1 *Ins2*^*Akita/+*^ and type 2 *Lepr*^*db/db*^ diabetic mice exhibited enhanced mitochondrial fission with altered cristae morphology (Fig. 3h–l and Extended Data Fig. 3a–j). However, overexpression of NDUFS4 restored the tubular and interconnected mitochondrial morphology as shown by improved mitochondrial aspect ratio, circularity, roundness, and ferret measurements in podocytes (Fig. 3h–l and Extended Data Fig. 3a–j). In addition, primary podocytes from diabetic *Ndufs4*^*podTg*^ mice also exhibited reduced mitochondrial ROS (Fig. 3m), and enhanced ATP production (Fig. 3n) compared to those from the diabetic *Ins2*^*Akita/+*^ mice.

### Ndufs4 overexpression prevents cristae remodeling.

**Extended Data Fig. 4 |:**
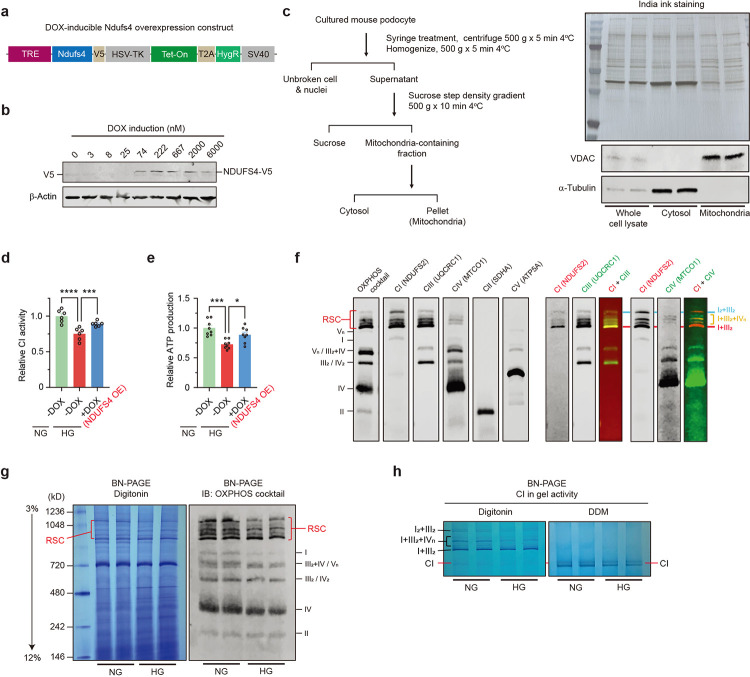
DOX-induced NDUFS4 OE in podocytes improves HG-induced mitochondrial remodeling. **a**, *PiggyBac* transposon vector containing a doxycycline (DOX)-inducible Ndufs4 (NDUFS4 OE) expression cassette. **b**, NDUFS4 expression induced by DOX at different concentrations. **c**, Procedure of mitochondrial isolation by sucrose step density gradient centrifugation using cultured podocytes (left panel), and immunoblot validation of mitochondrial purity in cytosol, and mitochondrial-enriched fraction (bottom right panel). Protein extracts from whole kidney was used as a control. India ink staining (upper right panel) showed the amounts of proteins loaded. **d**,**e**, Relative CI enzymatic activity in isolated mitochondria (**d**) and ATP production (**e**) in podocytes cultured under NG (5.5 mM) and HG (25 mM) for 48hrs with (NDUFS4 OE) or without DOX induction (normalized to NG levels; n=6 and n=8 replicates, respectively). **f**, Immunoblots of BN-PAGE of digitonin-solubilized mitochondria from DOX-induced NDUFS4 OE podocytes showing individual complexes as well as stoichiometry of respiratory supercomplexes (RSC). **g**, Coomassie staining (left panel) and immunoblot (right panel) of BN-PAGE analysis of digitonin-solubilized mitochondria isolated from podocytes cultured under NG and HG for 48hrs. **h**, CI in-gel activity of digitonin- and n-Dodecyl β-D-maltoside (DDM)-solubilized mitochondria from the same cells as in Extended Data Fig. 4g. RSC: respirasome supercomplexes. Roman numerals indicate RSC with defined stoichiometry of individual complexes. Data are presented as mean ± SEM (**d**,**e**) *P < 0.05, ***P < 0.001, ****P < 0.0001. One-way ANOVA with post-hoc Tukey’s test (**d**,**e**).

**Fig. 4 |:**
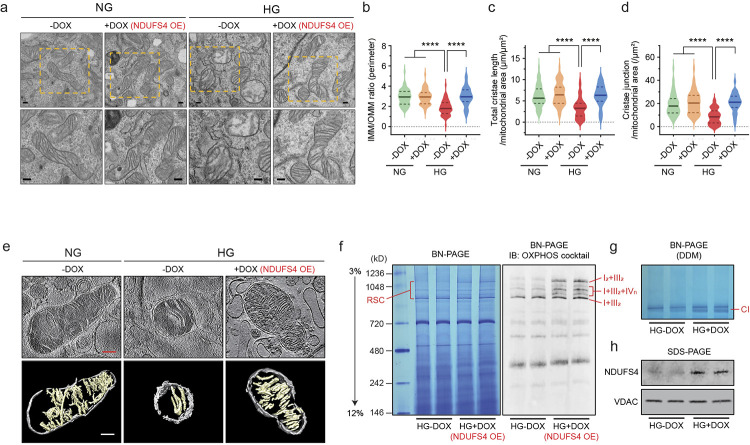
Cristae integrity and RSCs formation are restored by Ndufs4 overexpression. **a**-**d,** Representative TEM micrographs of cristae morphology (**a**) and quantitative analyses of cristae density (**b**-**d**) in podocytes cultured under NG (5.5 mM) or HG (25 mM) for 48hrs with DOX induction (NDUFS4 OE) or without DOX induction (n=60/group, scale bars: 200 nm). IMM: inner mitochondrial membrane, OMM: outer mitochondrial membrane. **e**, Cryo-ET analysis of purified mitochondria in DOX-inducible Ndufs4 transfected podocytes cultured under NG without DOX, HG without DOX, and HG with DOX induction (NDUFS4 OE). Slices through representative Cryo-ET tomograms are shown in upper panels and corresponding segmentations of characteristic features in lower panels (Scale bars: 200 nm). **f**, BN-PAGE of digitonin-solubilized mitochondria isolated from HG-DOX or DOX-inducible NDUFS4 (NDUFS4 OE). Left panel: Coomassie staining; right panel: Immunoblots with OXPHOS cocktail antibodies. RSC: respiratory supercomplexes. **g**, Representative CI in-gel activity of n-Dodecyl β-D-maltoside (DDM)-solubilized mitochondria isolated from the same cells as in Fig. 4f. **h**, Immunodetection of NDUFS4 after SDS-PAGE of mitochondria proteins in cells from Fig. 4f. VDAC was used as a loading control. Data are presented as median ± IQR (Bold line: median, and dot line: IQR). ***P < 0.001, ****P < 0.0001. Kruskal-Wallis with post-hoc Dunn’s test (**b**-**d**).

To further interrogate the causal link between Ndufs4 overexpression (OE) and mitochondrial reprogramming, we generated a *PiggyBac* transposon vector stably expressing a doxycycline (DOX)-inducible Ndufs4 expression (NDUFS4 OE) cassette (Extended Data Fig. 4a,b). Similar to our previous results with primary podocytes, we found enhanced mitochondrial fission and a significant cristae remodeling characterized by reduced cristae density in differentiated podocytes treated with HG (25 mM for 48 hrs), whereas cristae integrity was maintained in DOX-induced NDUFS4 OE in HG stress conditions, mainly resembling cristae morphology in NG conditions (5.5 mM for 48 hrs) (Fig. 4a–d). We also validated prevention of HG-induced reduced CI activity in mitochondria and decreased ATP production in NDUFS4 OE podocytes cultured under HG conditions (Extended Data Fig. 4c–e).

Since several publications have recently established the link between intact cristae morphology, the stability of respiratory supercomplexes (RSC), and mitochondrial morphology^30,31^, we argued that a possible relationship between NDUFS4 OE and improved cristae integrity could explain some of the beneficial effects of NDUFS4 OE on mitochondrial morphology. To this end, we first validated our observations using electron cryo-tomography (cryo-ET) of cristae structures and found substantial differences in cristae morphology consistent with a significant loss of cristae membranes in HG-exposed podocytes (Fig. 4e), whereas transfection of podocytes with NDUFS4 OE led to improved cristae integrity and mitochondrial morphology (Fig. 4e). To correlate these results with RSCs assembly, we examined the role of NDUFS4 OE on the abundance of intact RSCs in digitonin-treated mitochondrial samples by blue native polyacrylamide gel electrophoresis (BN-PAGE) ^32,33^. The abundance of intact RSCs on BN-PAGE gels was reduced in HG conditions (Extended Data Fig. 4f,g) whereas cultured NDUFS4 OE podocytes in HG media exhibited significantly higher protein abundance of RSCs (Fig. 4f). Western blot analysis of the same samples with OXPHOS cocktail antibodies provided similar results (Extended Data Fig. 4f,g). Consistent with these results, the CI in-gel activity as shown by reduction of nitro-blue tetrazolium in the presence of NADH, was reduced in podocytes treated with HG as compared to those treated with NG (Extended Data Fig. 4h), whereas NDUFS4 OE restored the CI in-gel activity even under HG conditions (Fig. 4g,h). Taken together, our findings suggest that NDUFS4 OE regulates not only cristae morphology but also RSCs assembly and mitochondrial dynamics in kidney podocytes.

### NDUFS4 interaction with cristae regulatory proteins.

**Extended Data Fig. 5 |:**
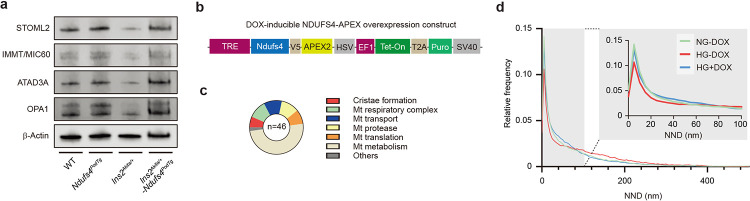
Doxycycline-inducible Ndufs4-APEX construct and NDUFS4-interactome. **a**, Immunoblotting of STOML2, IMMT/MIC60, ATAD3, and OPA1 in primary podocytes. β-Actin was used for loading control. **b**, *PiggyBac* transposon vector containing a doxycycline (DOX)-inducible NDUFS4-APEX expression cassette. **c**, The top 46 NDUFS4 associated mitochondrial proteins categorized into various groups according to their biological functions. Mt: mitochondrial. **d**, Trajectory of histograms for nearest neighboring distance (NND). Histograms show the fraction of the distance between NDUFS4 molecule and nearest neighboring STOML2 molecule.

**Fig. 5 |:**
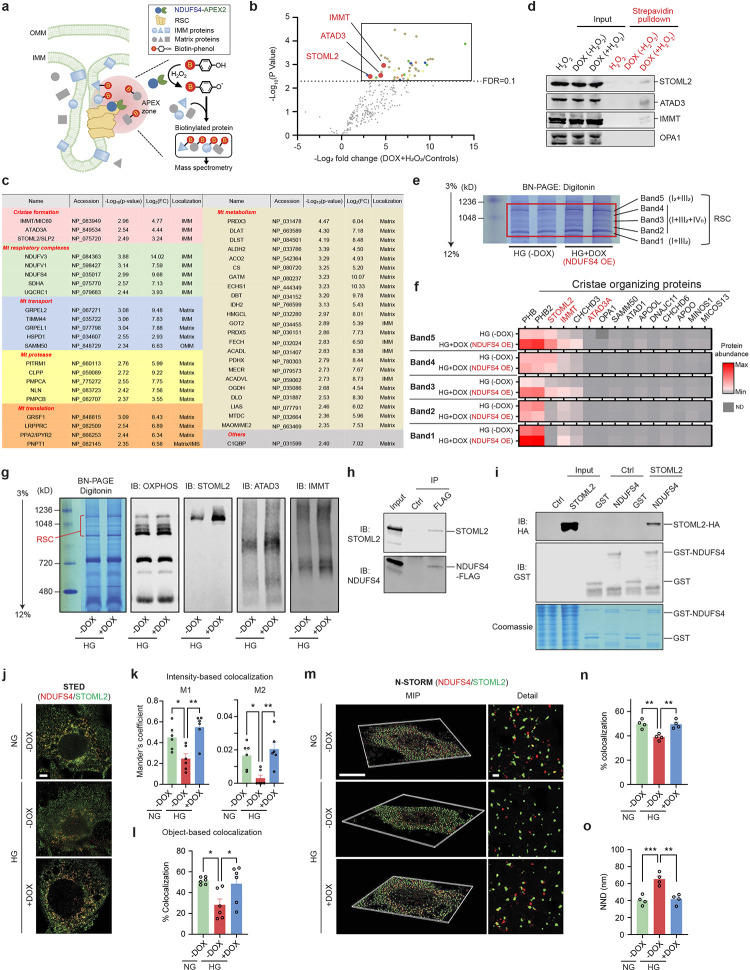
STOML2 interacts with NDUFS4. **a**, Schematic depiction of APEX2 proximity labeling followed by the LC-MS/MS analysis in podocytes transduced with a DOX-inducible NDUFS4-APEX2 chimeric construct. B: biotin, IMM: inner mitochondrial membrane, OMM: outer mitochondrial membrane, RSC: respiratory supercomplexes. **b**, Volcano plots of NDUFS4-associated mitochondrial proteins identified by LC-MS/MS analysis. The average values of NDUFS4-APEX2 transfected podocytes without H_2_O_2_ and those without DOX induction are used as controls. The dotted line indicates a cut off for significant change based on FDR *Q*< 0.1. **c**, NDUFS4 interactomes identified by the LC-MS/MS following APEX2 labeling. **d**, Immunoblotting of STOML2, ATAD3, and IMMT in whole cell lysates (Input) and streptavidin pulldown samples. OPA1 was used as a negative control. **e**, RSCs were excised (Band 1 to 5) from BN-PAGE, followed by LC/MS-MS analysis. **f**, Heatmap of cristae organizing proteins in the RSCs from podocytes cultured in HG with DOX induction (HG+DOX) or without (HG-DOX). **g**, Immunoblotting of OXPHOS, STOML2, ATAD3 and IMMT following BN-PAGE analysis as described in Fig. 5e. **h**, Co-IP of NDUFS4 and STOML2. HEK 293T cells were transiently transfected with Ndufs4-FLAG expression construct. Input was used as a positive control while IgG was used as a negative control (Ctrl). **i**, GST affinity pulldown assay in HEK293T cells overexpressing a STOML2-HA fusion protein. **j**, Representative images of NDUFS4 (red) and STOML2 (green) by STED analysis in cultured podocytes under the NG, HG, and HG plus NDUFS4 OE conditions (Scale bar: 5 μm). **k**, Intensity-based colocalization expressed by Mander’s coefficient. M1 and M2 indicate the coefficient for NDUFS4 overlapping STOML2 and STOML2 overlapping NDUFS4, respectively (n=6). M2 coefficient was substantially lower than M1 since STOML2 as compared to NDUFS4 is more widely distributed both in cytoplasm and mitochondria^48^. **l**, Object-based colocalization expressed by percent colocalization (n=6). **m**, Representative maximal intensity projection (MIP) images and detailed molecular images of NDUFS4 (red) and STOML2 (green) by STORM in cultured podocytes under the same conditions as shown in Fig. 5j. Scale bars: 10μm (left panel) and 500 nm (right panel). **n**, Percent colocalization based on the nearest neighboring distance (NND) <40 nm (n=4). **o**, Median NND (n=4). Data are presented as mean ± SEM. *P < 0.05, **P < 0.01, ***P < 0.001. One-way ANOVA with post-hoc Tukey’s test (**k**,**l**,**n**,**o**).

The regulatory effect of NDUFS4 on cristae morphology, RSCs integrity and mitochondrial dynamics raises several questions regarding the underlying protective molecular mechanism of NDUFS4 OE. It is known that cristae integrity is necessary for the proper spatial distribution of RSCs^34,35^. However, whether changes in ETC integrity could result in cristae remodeling is not well understood. We suspected that the effects of NDUFS4 on cristae remodeling could be through its interaction with one or several cristae regulatory proteins. To this end, we first assessed the abundance of some of the key cristae regulatory proteins by Western blots in podocytes from WT, *Ndufs4*^*podTg*^, diabetic *Ins2*^*Akita/+*^, and diabetic *Ndufs4*^*podTg*^ mice. The cristae regulatory proteins, including STOML2 (Stomatin-like protein 2), IMMT/MIC60 (Inner membrane mitochondrial protein), ATAD3A (ATPase family AAA domain containing 3A) and OPA1 (OPA1 mitochondrial dynamin like GTPase) were all reduced in the podocytes of diabetic *Ins2*^*Akita/+*^ mice (Extended Data Fig. 5a). Conversely, the abundance of these proteins was significantly higher in podocytes from diabetic *Ndufs4*^*podTg*^ (Extended Data Fig. 5a). We next adopted a proximity labeling approach to interogate a possible interaction of NDUFS4 with cristae regulatory proteins (Fig. 5a). To this end, we engineered a podocyte cell line that stably express a DOX-inducible NDUFS4-APEX2 chimeric protein (Extended Data Fig. 5b). Biotin-labeled proteins in proximity to NDUFS4-APEX2 activated with H_2_O_2_ were isolated using streptavidin-coupled beads and identified by LC-MS/MS. NDUFS4-APEX2 transfected podocytes without H_2_O_2_ activation or without DOX induction were used as controls. Out of 2152 proteins identified, 357 were mitochondrial proteins, and 46 of them were significantly enriched in DOX+H_2_O_2_ podocytes (Fig. 5b,c and Extended Data Fig. 5c). Among them, we found several cristae regulatory proteins, including STOML2, IMMT/MIC60, and ATAD3A (Fig. 5c and Extended Data Fig. 5c). The close proximity of NDUFS4 to the cristae regulatory proteins was further validated by immunoblot analysis using specific primary antibodies whereby the biotinylated STOML2, ATAD3A, and IMMT/MIC60 were efficiently pulled down by streptavidin beads when treated with H_2_O_2_ (Fig. 5d). Surprisingly, OPA1 was not a closely associated protein with NDUFS4 based on both the proximity labeling and strepavidin pulldown assays. Taken together, these findings suggest that the cristae regulatory proteins could potentially form complexes with NDUFS4. However, proximity labeling assay is unable to differentiate between a direct interaction or a mere close association. Additionally, it does not specify if the association of NDUFS4 with these proteins occurs in the context of individual complexes or RSCs. Consequently, we performed complexsome profiling on enriched mitochondrial samples from HG-treated cells and HG-treated NDUFS4 OE podocytes to explore whether the cristae forming proteins interact with NDUFS4 in the context of RSCs and therefore, comigrating within RSCs (Fig. 5e). We separated ETC complexes on BN-PAGE, sliced five distinct bands representing distinct RSCs (labeled 1–5) after Coomassie blue staining, and performed mass spectrometry (LC-MS/MS). A careful analysis of each band revealed that several cristae organizing proteins, including STOML2, ATAD3A and IMMT/MIC60, comigrate with RSCs, suggesting that they are in close association with RSCs structures (Fig. 5f). To further test whether STOML2, ATAD3A and IMMT/MIC60 are integrated within RSCs, we performed immunoblotting with cocktail antibodies against OXPHOS or cristae organizing proteins (Fig. 5g). We observed that whereas STOML2 was mainly colocalized with RSCs and its abundance was markedly increased with NDUFS4 OE, ATAD3 was not significantly colocalized with RSCs, and IMMT displayed a more wide-spread distribution within and outside of RSCs (Fig. 5g). Since STOML2 comigrated with RSCs in a NDUFS4 OE-dependent manner and considering our previous proximity labeling and pulldown assays, we decided to further pursue its role as a link between NDUFS4 OE and cristae integrity.

To further corroborate the physical interaction of NDUFS4 with STOML2, we employed a combination of biochemical and super-resolution imaging approaches. We first performed co-immunoprecipitation (Co-IP) experiments in HEK293T cells transiently transfected with NDUFS4-FLAG construct. Among pulled down proteins with FLAG antibody, both STOML2 and NDUFS4 were detected, but not with IgG antibody (Fig. 5h). We then performed a GST affinity pulldown assay *in vitro* in which a GST-NDUFS4 fusion protein was incubated with cell lysates transiently overexpressing a STOML2-HA fusion protein. Immunoblotting showed that STOML2 interacted with GST-NDUFS4, but not with GST control *in vitro,* consistent with a potential interaction between NDUFS4 and STOML2 (Fig. 5i).

To further establish whether NDUFS4 and STOML2 are spatially in close physical proximity, we employed two super-resolution imaging approaches, the stimulated emission depletion (STED) and the stochastic optical reconstruction microscopy (STORM) providing nanoscale spatial localization at a single-molecule resolution^36^. The STED super-resolution microscopy following dual immunostaining of podocytes with antibodies against NDUFS4 and STOML2 showed higher degree of colocalization between NDUFS4 and STOML2 in HG-treated NDUFS4 OE podocytes compared with podocytes cultured under HG conditions (Mander’s M1 coefficient=0.45±0.05 for NG-DOX, 0.25±0.05 for HG-DOX and 0.55±0.05 for HG+DOX; M2 coefficient=0.017±0.003 for NG-DOX, 0.003±0.002 for HG-DOX, and 0.021±0.004 for HG+DOX) (Fig. 5j,k). Similar results were confirmed in the analysis of colocalization based on the distance (Fig. 5j,k). Additional colocalization analyses of 3D images with single molecule super-resolution imaging obtained from STORM validated the spatial interaction between NDUFS4 OE and STOML2 (Fig. 5m). Specifically, among molecules with the inter-molecular distance <500 nm (Extended Data Fig. 5d), podocytes under the HG condition exhibited a significantly longer distance between NDUFS4 and the nearest neighboring STOML2, with a median nearest neighboring distance (NND) that was also significantly longer compared to podocytes under the NG or HG-treated NDUFS4 OE conditions (% colocalization=38.9±1.3% for HG-DOX *vs.* 49.8±1.8% for NG and 49.5±1.8% for HG+DOX; NND= 65.3±3.6 nm for HG-DOX *vs.* 40.3±2.9 nm for NG and 41.6±3.0 nm for HG+DOX) (Fig. 5n,o). These findings suggest that within each mitochondrion, Ndufs4 and STOML2 have the highest proximity in NG or with Ndufs4 overexpression in HG conditions. In contrast, they exhibit the least spatial proximity in HG conditions.

### STOML2 is required for NDUFS4-mediated RSCs assembly and cristae integrity.

**Fig. 6 |:**
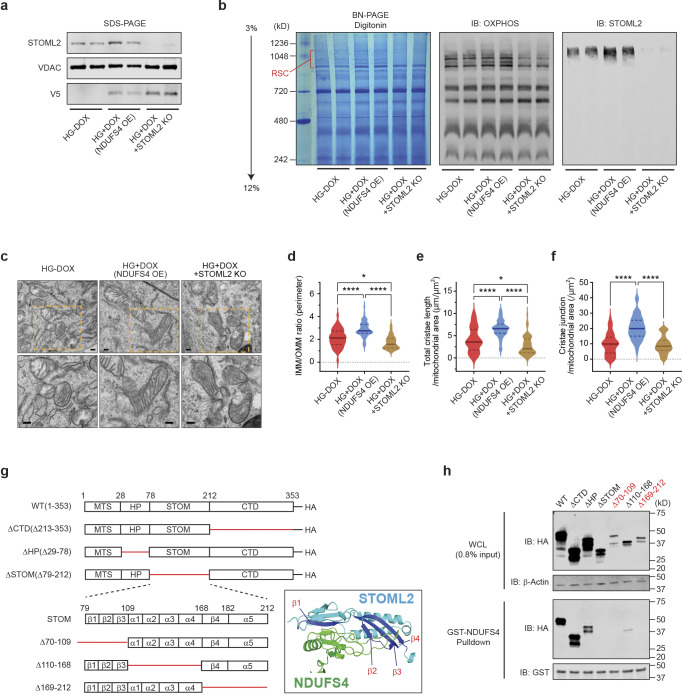
STOML2 is essential for NDUFS4-mediated improvement of RSCs formation and cristae remodeling. **a**, Immunoblot analysis of indicated proteins in NDUFS4 overexpression stable podocytes cultured in high glucose (HG) without (HG+DOX) or with (HG+DOX+STOML2KO) STOML2 CRISPR targeting; parental podocyte without Ndufs4 transduction was used as a control (HG). NDUFS4 was blotted with V5 tag antibody. **b**, BN-PAGE of digitonin-solubilized mitochondrial proteins from the same cells as shown in Fig. 5A stained with Coomassie or immunoblotted (IB) with OXPHOS cocktail or STOML2 antibodies. RSC: respiratory supercomplexes, DOX: doxycycline. **c**, Representative TEM micrographs of mitochondria in cells described in Fig. 6a, and cristae density measurements by IMM/OMM ratio (**d**), total cristae length (**e**) and cristae junction (**f**), (n=60 mitochondria, scale bars: 200 nm). IMM: inner mitochondrial membrane, OMM: outer mitochondrial membrane. **g**, Structure of mouse STOML2 wild type (WT) and deletion mutant constructs engineered with HA-tag at the C-terminus. MTS: mitochondrial targeting sequence. HP: hydrophobic hairpin. STOM: stomatin. CTD: C-terminal coiled-coil domain. β1–4: β1–4 sheets in STOM domain. α1–5: α1–5 helices in STOM domain. Protein-protein docking simulation of human STOML2 (blue) and NDUFS4 (green; in the context of human respirasome). **h**, GST pull-down assay from HEK293T cells overexpressing indicated STOML2-HA fusion proteins or deletion mutants. WT: full length wild type, ΔCTD: deletion mutant of C-terminal domain, ΔHP: deletion mutant of hydrophobic hairpin domain (Δ29–78), ΔSTOM: deletion mutant of stomatin domain (Δ79–212), Δ70–109: β1–3 sheets mutant, Δ110–168: α1–4 helices mutant, Δ169–212: β4 sheet and α5 helix mutant. Data are presented as median ± IQR (Bold line: median, and dot line: IQR). *P < 0.05, **** *P* < 0.0001. Kruskal-Wallis with post-hoc Dunn’s test (**d**-**f**).

To investigate the functional impact of the interaction between STOML2 and NDUFS4 on RSC remodeling, we used STOML2 knockout (KO) podocytes in the presence or absence of NDUFS4 OE. After verifying deletion of STOML2 by CRISPR genome editing (Fig. 6a), we performed BN-PAGE analysis followed by immunoblotting. Both Coomassie staining and immunoblotting using OXPHOS cocktail antibodies showed an increase of RSCs abundance in NDUFS4 OE podocytes. However, this increase was markedly reduced in STOML2 KO cells despite NDUFS4 OE (Fig. 6b). We also observed that HG reduced the mitochondrial cristae density in podocytes, but NDUFS4 OE restored the cristae structure and abundance in HG media and improved mitochondrial morphology (Fig. 6c–f). In the NDUFS4 OE podocytes in which the STOML2 was deleted, however, the cristae restoration was no longer observed and mitochondrial morphology was distorted in HG conditions (Fig. 6c–f). Thus, these data suggest that STOML2 is an essential component of the NDUFS4 OE-mediated improved cristae integrity, RSC assembly, and mitochondrial morphology.

**Extended Data Fig. 6 |:**
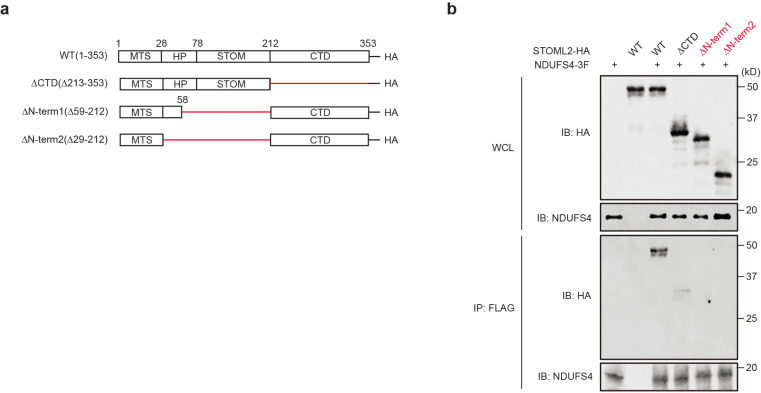
NDUFS4 binds STOML2 at N-terminus domain. **a**, Structure of mouse STOML2 WT and deletion mutant constructs engineered with HA-tag at the C-terminus. MTS: mitochondrial targeting sequence. HP: hydrophobic hairpin. STOM: stomatin. CTD: C-terminal coiled-coil domain. ΔCTD: deletion mutant of c-terminus mutant (Δ213–353). ΔN-term1: deletion mutant of N-terminus 1 (Δ59–212). ΔN-term2: deletion mutant of HP and STOM (Δ29212). **b**, Co-IP assay of NDUFS4 and STOML2 in HEK293T cells transfected with NDUFS4-FLAG and indicated STOML2-HA constructs shown in Extended Data Fig. 6a, using FLAG antibody-conjugated beads. WCL: whole cell lysate.

The full-length mouse STOML2 protein is 353 amino acids long and contains four functional domains: the N-terminal mitochondrial-targeting sequence (MTS), a hydrophobic hairpin (HP) domain, a conserved stomatin (STOM) domain, and the C-terminal coiled-coil domain (CTD) (Fig. 6g). To address how NDUFS4 interacts with STOML2, we created a series of STOML2 deletions and observed that the C-terminal deletion mutant was co-immunoprecipitated with FLAG tagged-NDUFS4, but not the other mutants at the N-terminal domain which included HP and STOM domains, suggesting that the N-terminal domain of STOML2 is the key domain for NDUFS4 binding (Extended Data Fig. 6a,b). We next generated additional mutants harboring the N-terminal domain and performed GST-NDUFS4 pull-down assay (Fig. 6g). While deletion mutants of C-terminal (ΔCTD (Δ213–353)), HP domain (ΔHP(Δ29–78)), and the α1–4 helices of the STOM domain (Δ110–168) did not prevent binding to NDUFS4, complete deletion of STOM domain (ΔSTOM (Δ79–212)), as well as deletions of β1–3 sheets (Δ70–109) and β4 sheet plus α5 helix (Δ110–168) of STOM domain prevented binding of STOML2 to GST-NDUFS4 (Fig. 6g,h), indicating that NDUFS4 could bind to STOML2 at the regions of β1–4 sheets and α5 helix in the STOM domain. This result is consistent with a molecular docking simulation analysis which showed two regions, 70–109 and 169–212, characterized by four β-sheets secondary structures in STOM domain (Fig. 6g), have a high probability to interact with NDUFS4 within the RSC structure^37^. Taken together, these data collectively suggest that two regions of the β-pleated sheet structures in STOM domain of STOML2 are crucial for its binding to NDUFS4.

## DISCUSSION

The mitochondrial ETC harnesses the chemical energy of nutrients in the form of high-energy electrons to generate an electrochemical proton gradient leading to the reduction of molecular oxygen to water. Beyond its role on the mitochondrial respiration, electron flow across inner mitochondrial membrane is also crucial for the bioenergetics properties of mitochondria through synthesis of ATP and critically involved in the biosynthetic and signaling properties of mitochondria. Importantly, ETC dysfunction has been associated with several human pathologies^23–26^. However, the impact of impaired ETC assembly and the link between ETC, RSCs formation, and cristae integrity in the pathogenesis of DKD remained unknown.

The findings of this study provide a mechanistic link between ETC remodeling in podocytes and the pathogenesis of DKD, a major complication of diabetes. Using an integrated *in vitro* and *in vivo* experimental approach, we find a causal link between NDUFS4 deficiency, an accessory component of CI of ETC, in podocytes and the pathogenesis of DKD *in vivo*. These findings represent a major paradigm shift in the current management of DKD by suggesting that targeting ETC remodeling could be a promising approach for developing therapies to mitigate the progression of DKD.

**Fig. 7 |:**
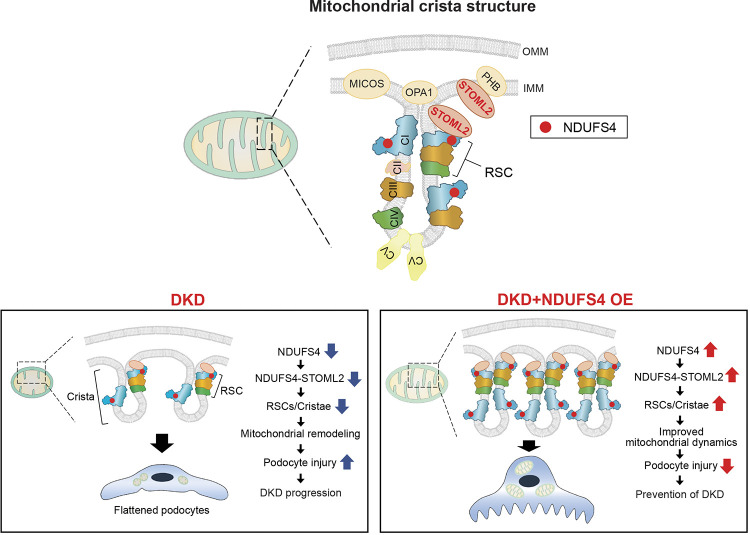
Schematics of NDUFS4-mediated cristae remodeling in DKD. Normal crista structure (top) and proposed model depicting the remodeling of ETC in podocytes from diabetic condition (lower left), and how NDUFS4 OE restored the mitochondrial morphology and function (lower right). DKD: diabetic kidney disease, IMM: inner mitochondrial membrane, OMM: outer mitochondrial membrane, RSC: respiratory supercomplexes.

Our findings also uncover the central role of ETC integrity as a defining feature of mitochondrial dysfunction and a powerful regulator of cristae remodeling and mitochondrial dynamics in the diabetic milieu. We discovered an unexpected role of Ndufs4 as a major culprit in maintaining cristae morphology and mitochondrial shape beyond its previously well-established role in CI assembly and stabilization^38,39^. Our study also highlights the benefits of rescuing Ndufs4 deficiency on mitochondrial dysfunction and DKD progression (Fig. 7).

While previous studies have identified the critical role of cristae shaping proteins, including STOML2, in cristae remodeling and RSCs assembly^34,40,41^, the regulatory role of ETC integrity on cristae structure and the interaction between NDUFS4 and cristae shaping proteins remained unknown. A first hint that NDUFS4 deficiency could have a key role in defining mitochondrial dysfunction in DKD came from our initial comparative proteomic profiling revealing a consistent downregulation of several subunits of CI in diabetic podocytes. We reasoned that one possible explanation for the CI remodeling in podocytes could be an initial adaptive mechanism to the diabetic environment. However, as hyperglycemia persists, these chronic changes might have become maladaptive and pathogenic in nature resulting in biochemical and structural alterations in mitochondria. To this end, we argued that forced expression of the NDUFS4 subunit, consistently downregulated in the podocytes of the type 1 and type 2 diabetic mice as well as in the glomeruli of the DKD patients, might overcome the mitochondrial maladaptation in DKD and provide significant insights into molecular mechanisms of its progression. Indeed, we found that overexpression of the NDUFS4 in podocytes improved mitochondrial respiration and CI activity and prevented mitochondrial fragmentation in podocytes of diabetic mice. We also linked NDUFS4 overexpression with the protection of both RSC assembly and cristae morphology.

How does NDUFS4 overexpression restore cristae organization and mitochondrial dynamics? We had previously shown that enhanced mitochondrial fission is implicated in the diabetes-induced mitochondrial dysfunction in podocytes^28,29^. However, the role of ETC integrity on cristae morphology and mitochondrial fission was incompletely understood. Our qualitative and quantitative approaches in this study including tomography clearly suggest aberrant cristae morphology and a significant loss of their native lamellar morphology. Remarkably, we find that these alterations in cristae structure were significantly improved with the forced expression of Ndufs4. By coupling proximity labeling, streptavidin pulldown assays, complexsome profiling, and super-resolution imaging approaches, we identified a possible interaction between STOML2, a 39 kDa cristae shaping protein, and NDUFS4 in the context of improved RSCs assembly as the main explanation for the NDUFS4-mediated improvement in cristae morphology. We further validated this interaction and found that two regions of the β-pleated sheet structures in STOM domain of STOML2 are crucial for its binding to NDUFS4. These observations complement previous publications on STOML2^40^ and other cristae shaping proteins including OPA1^34^ and MICOS^41^, indicating that beyond their cristae regulating properties, these cristae shaping proteins also regulate RSCs integrity and mitochondrial dynamics. However, while the effect of cristae organizing proteins on cristae integrity has clearly been established, our findings uncover a novel role of Ndufs4 in regulating cristae and RSC integrity and ultimately mitochondrial morphology and function. We speculate that overexpression of Ndufs4 subunit stabilizes and improves its interaction with STOML2 leading to proper cristae formation, RSCs assembly and improved mitochondrial respiration and dynamics in the diabetic milieu.

The NDUFS4 subunit is localized between the N-and Q-module and it is known that pathogenic mutations in the nuclear DNA-encoded *NDUFS4* gene, the most extensively studied mutations in CI subunit, cause a severe form of Leigh-like Syndrome in pediatric populations, a rare and heterogeneous disorder that affects central nervous system^26,42^. Similarly, mice lacking full length NDUFS4 protein develop Leigh (like) disease with postnatal lethality at ~50 days^43^. Several tissue-specific Ndufs4 knockout mouse models are also developed to further examine the role of NDUFS4 as a model to study mitochondrial diseases^44–47^. Comprehensive analysis of cells derived from these mice revealed that tissue-specific NDUFS4 mutations were commonly associated with increased ROS, altered mitochondrial ATP homeostasis and mitochondrial morphology^44,47^. However, it is important to emphasize that our data suggest that the diabetes-induced ETC dysfunction is not exclusively related to NDUFS4 deficiency and the abundance of several other subunits of CI were altered in the podocytes of diabetic mice. Future studies are needed to carefully examine the role of other subunits of CI on progression of DKD.

Taken together, our gain-of-function approach shed new light on the otherwise unappreciated crucial role of Ndufs4 in maintaining the structural integrity of cristae and mitochondrial function in podocytes in the diabetic environment which is translated into physiological restoration of podocyte function in diabetic mice *in vivo*.

Our study has unraveled many unexpected aspects of pathobiology of NDUFS4 in podocytes and its role in the progression of DKD, however, our findings also raise several important questions that remain to be fully addressed. For example, what upstream signaling pathways are required to initiate the cascade of events that lead to reduced expression of NDUFS4 in podocytes in DKD? Furthermore, additional experiments are needed to determine the extent of NDUFS4 deficiency in other cells and tissues. It would also be meaningful to examine the role of other subunits of CI on progression of DKD. The interplay between NDUFS4 and STOML2 is also complex and further studies are needed to test whether STOML2 can be regarded as a specific and necessary binding partner of NDUFS4 in maintaining cristae structure. Indeed, to better understand the pathobiology and structural integrity of NDUFS4 in the diabetic milieu, it would be important to understand the molecular relationship between NDUFS4 with other cristae organizing proteins as they relate to the recruitment of the supercomplexes and improved mitochondrial dynamics. Finally, one important question is whether our findings are relevant to humans. Our data indicates that reduced levels of glomerular NDUFS4 expression correlate with albuminuria and eGFR in subjects with DKD. Importantly, we find that NDUFS4 staining in glomeruli is progressively reduced with worsening of DKD histology suggesting that Ndufs4 may play an important role in progression of DKD. However, mice and humans present major differences that might influence the dynamics of the events described in this study and further studies are required to validate our results in individuals with DKD.

In summary, our study suggests that reduced levels of NDUFS4 expression leads to compromised CI and RSCs formation with a significant effect on bioenergetic capacity, cristae integrity, and mitochondrial morphology of podocytes promoting DKD progression. We discovered that forced expression of NDUFS4 in the diabetic environment, however, leads to significant improvement in RSCs assembly and cristae and mitochondrial morphology mitigating DKD progression. We propose that strategies aimed at improving NDUFS4 expression in DKD could emerge as a paradigm shifting intervention for ameliorating progression of DKD.

## Methods

### Mouse models

All animal studies were reviewed and approved by the Institutional Animal Care and Use Committee of the University of Texas at MD Anderson Cancer Center and conducted according to the institutional and the US National Institutes of Health guidelines. All mice were maintained in a temperature-controlled environment (22°C) under a 12-hrs light/dark cycle with free access to chow and water. All mice were maintained with ad Librium access to food and water under the ambient temperature of 23°C. Type 1 and type 2 diabetic mice (*Ins2*^*Akita/+*^ on C57BL/6J background and *Lepr*^*db/db*^ on C57BLKS/J background) were obtained from Jackson Laboratories (Stock Nos. 003548 and 000642). Urine samples were used to measure albumin and creatinine concentration using mouse albumin ELISA kit (Ethos Biosciences) and QuantiChrom creatinine assay kit (Bioassay Systems), respectively. Hemoglobin A1c was measured by HbA1c Kit (Crystal Chem).

The Podocyte-specific Ndufs4 transgenic mice were generated using a podocyte-specific transgenic Ndufs4 construct under the control of a human podocin promoter driving a murine Ndufs4 cDNA followed by a WPRE and a human growth hormone polyadenlyation signal. Transgenic cassette was flanked by chicken hypersensitivity site 4 (HS4) insulator. The DNA construct was restriction digested, purified and microinjected into pronuclei of C57BL/6J embryos by the BCM Genetically Engineered Rodent Models Core. Transgenic mice genotyping was performed using genomic DNA isolated from mouse tails by PCR with the following primers: 5’-ACTCCACAGGGACTGCGCTC-3’; 5’-CCGAGTCTGGTTGTCTGCCA-3’. A 217-bp DNA fragment can be amplified only from the Ndufs4 transgenic mice.

### Cell lines and maintenance

Conditionally immortalized mouse podocytes were a kind gift from Jochen Reiser (Rush University, Chicago, IL). In brief, cells were cultured at 33°C in RPMI (Corning) containing 10% FBS (GenDepot), antibiotic antimycotic solution (Corning), and 20 U/ml mouse recombinant IFN-γ (Sigma) to enhance expression of a thermosensitive T antigen. The cells were differentiated at 37°C in DMEM (Corning) supplemented with 5% FBS and antibiotic antimycotic solution without IFN-γ on collagen type I (Gibco) coated dishes for 7–12 days. For imaging, differentiated cells were trypsinized, dissociated, and plated onto collagen type I coated coverslips. Podocytes prepared for experiments involving high glucose conditions (HG, 25mM) were serum deprived for 24 hours prior to addition of HG. Control cells were cultured with normal glucose (NG, 5.5mM). For primary cultured podocytes, isolated podocytes from WT and *Ndufs4*^*podTg*^ mice were cultured with DMEM containing NG, 5% FBS, and antibiotic antimycotic solution, while those from *Ins2*^*Akita/+*^ and *Ins2*^*Akita/+*^*;Ndufs4*^*podTg*^ mice were cultured with DMEM containing HG, 5% FBS, and antibiotic antimycotic solution just before each experiment.

### Cloning and genome editing

Mouse Ndufs4 cDNA fused with 3’-V5 tag was amplified from mouse tail genomic DNA by PCR, then subcloned into a *PiggyBac* transposon-based vector engineered with TRE promoter and hygromycin selection marker^49^. Stoml2 cDNA (NM_023231) was purchased from OriGene. Stoml2 deletion mutants were generated using Q5 Site-Directed Mutagenesis Kit (New England Biolabs) and verified by sequencing. Gene editing of Stoml2 was carried out using evolved Cas9^50^ in an engineered *PiggyBac* system with puromycin selection. The gRNA pair targeting exon 1 and exon 2 of Stoml2 in CRISPR is: GTGGGAAATGCTGGCGCGCGCGG and TCACCGGTTCCAGGATCCGGTGG.

### Human kidney biopsy samples

Human kidney biopsy samples were obtained from Okayama University Hospital in Okayama, Japan. The protocol was approved by the Institutional Review Board of the Ethics Committee of Okayama University Hospital and registered with the University Hospital Medical Information Network (UMIN) (identification number: UMIN000046398). For the control group, we analyzed kidney biopsy samples at the time of kidney transplantation from 9 donor participants. DKD was classified and histological scores were determined according to the criteria of the Renal Pathology Society^27^. For immunohistochemistry, antigen retrieval was performed by heating sections in 10 mM citrate buffer (pH 6.0) in a microwave oven. Reactions with endogenous peroxidases and proteins were blocked by incubation with 0.3% H_2_O_2_ diluted in methanol and serum-free protein blocking solution (Dako). Then, tissue was incubated with rabbit anti-NDUFS4 primary antibody (Abcam, ab137064) overnight at 4°C. Goat anti-rabbit IgG HRP Polymer (Vector Laboratories, MP-7451) was used as the secondary antibody and peroxidase activity was visualized with a liquid diaminobenzidine substrate (Vector Laboratories). Image J was used for the quantification of NDUFS4 intensity in each glomerular tuft area. Gene expression data were extracted from Nephroseq database version 5 (https://www.nephroseq.org). Glomerular transcriptomic data were analyzed using Ju CKD Glom dataset.

### Doxycycline (DOX)-inducible transient Ndufs4 expression

A DOX-inducible Ndufs4 overexpression construct, based on a reverse tetracycline-controlled transactivator (rtTA) and tetracycline-responsive element promoter (TRE), was engineered in the PiggyBac transposon system. For experiments, DOX at a concentration of 200 nM was used for 48–72hrs.

### RNA extraction and real time qRT-PCR

Cells were homogenized in TRIZOL (Invitrogen), and total RNA was purified using PureLink^™^ RNA Mini Kit (Invitrogen) according to the manufacture’s protocols. Real-time quantitative RT-PCR (qRT-PCR) was performed using the SYBR green dye (Applied Biosystems) with a StepOnePlus Real-Time PCR System (Applied Biosystems). Fold changes of gene expression was normalized by housekeeping genes and analyzed using the ΔΔCT method. The specific primers for target gene used in this study are listed in Supplementary Table 2.

### SDS-PAGE

Western blot assays were performed as described previously^28^. In brief, cells or purified mitochondria were resuspended in RIPA buffer (TEKnova) containing 1% protease inhibitor cocktail (Sigma). Protein concentration was determined using BCA protein assay (Pierce). To analyze mitochondrial proteins, lysates were heated at 42°C for 5 minutes in laemmli sample buffer (Bio-Rad), but other proteins were heated at 95°C for 5 minutes in the same buffer. 10–30 μg protein lysates were loaded onto 4–20% gradient SDS PAGE (Bio-Rad) and transferred to PVDF membranes (Roche). Membranes were probed with the primary antibodies followed by washing and adding the fluorescent secondary antibodies. Antibody-antigen reaction profiles were visualized and quantified by Odyssey XF Imager (LI-COR). Antibodies are summarized in Supplementary Table 3.

### Podocyte and tubular cell isolation

Podocyte and tubular cell isolation from mouse kidneys were performed as previously described with slight modifications^51,52^. In brief, podocytes were *ex vivo* selected by biotin-labeled anti-Kirrel3 and podocalyxin antibodies (R&D Systems BAF4910 and R&D Systems BAF1556, respectively), then isolated using magnetic, streptavidin labeled Dynabeads (Thermo Fisher). To isolate tubular cells, dissected and minced kidneys were digested with collagenase type II in RPMI media for 30 mins at 37°C. Cells were sieved first through a 100 μm nylon mesh, then through a 40 μm nylon mesh, followed by centrifugation at 500 g for 10 min. The pellet was resuspended in red blood cell lysis buffer (R&D Systems) and incubated on ice for 10min. After centrifuge at 500 g for 10 min, cells were resuspended in RIPA buffer (TEKnova) containing 1% protease inhibitor cocktail (Sigma) and stored at −80°C freezer for experiments.

### Mitochondrial isolation

Mitochondrial isolation from tissue was performed using Percoll density gradient centrifugation^53^. Mouse isolated podocytes were resuspended in mitochondrial isolation buffer (MIB1: 10 mM HEPES, 250 mM Sucrose, and 1 mM EDTA, pH 7.4, at 4°C), and homogenized by a glass homogenizer, followed by centrifuging homogenate at 1,300 g at 4°C for 3 min. After two cycles of homogenization and centrifugation, the pooled supernatant was centrifuged at 21,000 g at 4°C for 10 min. The resultant pellet was resuspended with 15% Percoll in MIB1 followed by purification by Percoll density centrifugation using a stepwise density gradient of 40%, 23%, and 15% Percoll in MIB1, at 30,700 g at 4°C for 5 min. Mitochondria accumulating at the interface between the 23% and 40% were collected, washed with MIB1, and centrifuged at 16,800 g at 4°C for 10 min. Mitochondrial pellet was resuspended with MIB1 and centrifuged at 7,000 g at 4°C for 10 min. Purified mitochondria was resuspend with MIB1, snap-froze in liquid nitrogen, and stored at −80°C freezer for experiments. Mitochondria from cultured podocytes were isolated using Sucrose step density gradient centrifugation method with some modifications^54^. In brief, cells were resuspended in another mitochondrial isolation buffer (MIB2: 3 mM HEPES, 210 mM Mannitol, 70 mM Sucrose, and 0.5 mM EDTA, 1 mM MgCl_2_, pH 7.4, at 4°C), and homogenized using a syringe with a 27.5 G needle. Homogenate was centrifuged at 500 g at 4°C for 5 min, and the resultant pellet was homogenized with MIB2 by a glass homogenizer, followed by the centrifugation at 500 g at 4°C for 5 min. After the supernatants were combined, the pooled supernatant was carefully added on the top of the same amount of 340 mM sucrose in a tube and centrifuged 500 g at 4°C for 10 min with low acceleration/deceleration. After the centrifugation, the top layer (mitochondrial fraction) was transfer to the fresh tube and centrifuged at 10,000 g at 4°C for 10 min. Pellet was resuspended with MIB2 and centrifuge at 7,000 g at 4°C for 10 min. Purified mitochondria was resuspend with 300 mM sucrose, snap froze in liquid nitrogen, and stored at −80°C freezer for experiments.

### Mitochondrial respiratory assay

Mitochondrial oxygen consumption rates were measured in cultured primary podocytes using a Seahorse Bioscience XFe-96 Analyzer according to the manufacturer’s instructions (Agilent Technologies). The optimal cell density for podocytes and the concentration of different drugs were previously described^51^. In brief, primary podocytes were seeded in 96-well culture plates coated with collagen I (0.1 μg/ml), and the following drugs were injected: oligomycin (2 μM), FCCP (2 μM), and rotenone (0.5 μM) and antimycin A (0.5 μM). Data were normalized using the CyQUANT assay kit (Invitrogen) and analyzed using Wave (version 2.6.0, Agilent) and Prism software packages (version 9, Graphpad). For the evaluation of rotenone sensitivity, podocytes were treated with different concentrations of rotenone (10 nM, 50 nM, 100 nM, and 250 nM) after stimulating with FCCP (2 μM). In each sample, dose-response curve was individually developed and the rotenone IC_50_ value was calculated to compare the sensitivity to CI inhibition.

### ATP and ROS measurement

Intracellular ATP levels and DNA concentration in each sample were measured using CellTitre-Glo 2.0 Cell Viability Assay Kit (Promega) and CyQUANT Cell proliferation Assay Kit (Molecular Probes), respectively, according to the manufacturer’s instructions. Data were expressed as the ratio of ATP concentration to DNA concentration. Mitochondrial ROS levels in primary podocyte were assessed using 5 μM MitoSOX Red mitochondrial superoxide indicator (Thermo Fisher) according to the manufacturer’s instructions. The intensity of MitoSOX Red was analyzed by flow cytometry as previously described^28^.

### Blue-Native PAGE

Mitochondrial enriched fractions were centrifuged at 10,000 g at 4°C for 5min, and the mitochondrial complexes were solubilized by digitonin (8 μg/μg mitochondrial protein, Sigma) or DDM (3 μg/μg mitochondrial protein, Thermo Fisher) with 4X NativePAGE sample buffer (Thermo Fisher). After centrifugation at 20,000 g for 10min at 4°C to remove insoluble material, Commassie G-250 was added to be the final concentration of one-fourth the detergent concentration. Next, 15 μg of proteins were run on a pre-cast 3%−12% gradient Native PAGE Bis-Tris Gel (Invitrogen) at 150 V at 4°C for 30min with Cathode Buffer B (50mM Tricine, 7.5mM imidazole, 0.02% Commassie G-250, pH 7.0), followed by 2.5hrs at 250V at 4°C with 1/10 Cathode Buffer B (50mM Tricine, 7.5mM imidazole, 0.002% Commassie G-250, pH 7.0) as previously described^32,55^.

### CI activity

CI activity in tissue sections was assessed by NADH diaphorase staining using kidney frozen sections from mice based on the previous report^43^. NADH oxidoreductase was assayed by incubating kidney sections in 50 mM Tris-HCl (pH 7.4), 0.8 mg/ml NADH (Sigma), and 1 mg/ml nitro blue tetrazolium (NBT, Sigma) for 1hr at RT. After washing in distilled water three times, samples were washed with 3 exchanges of the 30, 60, 90% acetone solutions in increasing then decreasing concentration to remove unbound NBT. After a rinse in distilled water, slides were mounted with the aqueous mounting medium. The intensity of CI activity was measured using image J. To assess in-gel activity for CI, BN gels were incubated in the assay buffer consisting of 5 mM Tris-HCl (pH 7.4) with 0.1 mg/ml NADH (Sigma) and 2.5 mg/ml NBT (Sigma) for 15 min at RT as described previously^32^.

### Scanning electron microscopy

Scanning electron microscopy was conducted as previously reported^29^. In brief, tissue samples fixed in solutions containing 3% glutaraldehyde plus 2% paraformaldehyde in 0.1 M cacodylate buffer (pH 7.3) were washed with 0.1 M cacodylate buffer (pH 7.3), postfixed with 1% cacodylate buffered osmium tetroxide (OsO4), washed with 0.1 M cacodylate buffer, then in distilled water. Afterwards, the samples were sequentially treated with Millipore-filtered 1% aqueous tannic acid, washed in distilled water, treated with Millipore-filtered 1% aqueous uranyl acetate, and then rinsed thoroughly with distilled water. The samples were dehydrated with increasing concentrations of ethanol, then transferred to increasing concentrations of hexamethyldisilazane (HMDS) and air dried overnight. Samples were mounted on to double-stick carbon tabs (Ted Pella), which have been previously mounted onto glass microscope slides. The samples were then coated under vacuum using a Balzer MED 010 evaporator (Technotrade International) with platinum alloy for a thickness of 25 nm, then immediately flash carbon coated under vacuum. The samples were transferred to a desiccator for examination. Samples were examined/imaged in a JSM-5910 scanning electron microscope (JEOL) at an accelerating voltage of 5 kV.

### Transmission electron microscopy

Transmission electron microscopy (TEM) was performed as previously described^29,56^. Tissue samples were fixed with a solution containing 3% glutaraldehyde plus 2% paraformaldehyde in 0.1 M cacodylate buffer (pH 7.3), then washed in 0.1 M sodium cacodylate buffer and treated with 0.1% Millipore-filtered cacodylate buffered tannic acid and postfixed with 1% buffered osmium. Cultured podocytes were fixed with a solution including 0.5% glutaraldehyde plus 2% paraformaldehyde. Fixed samples were washed in 0.1 M sodium cacodylate buffer (pH 7.4) and treated with 0.1% Millipore-filtered cacodylate buffered tannic acid, postfixed with 1% OsO4/1.5% potassium ferrocyanide (KFeCN6), and stained en bloc with 1% Millipore-filtered uranyl acetate. The samples were polymerized in a 60°C oven for approximately 3 days. Ultrathin sections were cut in a Leica Ultracut microtome (Leica), stained with uranyl acetate and lead citrate in a Leica EM Stainer, and examined in a JEM 1010 transmission electron microscope (JEOL) at an accelerating voltage of 80 kV. Digital images were obtained using AMT Imaging System (Advanced Microscopy Techniques).

### Mitochondrial morphology

Mitochondrial morphological measurement was performed as previously described^29^. Briefly, mitochondrial aspect ratio was defined as the major and minor axes of the ellipse expressed as a fraction. Circularity was 4π x (mitochondrial area (Am) per [perimeter (Pm)]^2^), and roundness was (4 x Am)/ (π x [major axis]^2^). Feret was the longest distance between any two points along mitochondrial perimeter.

### Cristae morphological assessment

A total of 60 mitochondria in TEM micrographs were analyzed using Image J. To quantify mitochondrial cristae abundance, inner/outer mitochondrial membrane perimeter ratio, total cristae length per mitochondrial area, and cristae junction number per mitochondrial area were measured as previously described^57,58^.

### Immunofluorescence staining in cells

Podocytes were washed with cold PBS, fixed in 4% formaldehyde, and permeabilized with 0.1% Triton X-100 (Acros). Cells were blocked in 1% BSA (Jackson Immuno-research), 50 mM Tris pH 7.6, 155 mM sodium chloride (TBS), 0.1% Triton-X-100. The cells were incubated overnight at 4°C with appropriate primary antibodies in blocking buffer. Coverslips were washed 3 times in TBS and incubated with appropriate secondary antibodies in blocking buffer for 1–2 hrs at RT. Coverslips were washed 3 times in TBS and mounted onto slides. Images were captured by FV1200 MPE confocal microscope (Olympus). Quantification was carried out using Image J. Antibodies and dyes used in this experiment are summarized in Supplementary Table 3.

### Mitochondrial proteome and complexsome profiling

Mitochondria enriched fractions isolated from podocytes were digested in a buffer containing 50 mM ammonium bicarbonate with LysC enzyme for 2 hrs at room temperature followed by Trypsin enzyme digestion at 37°C overnight. The digestion was neutralized by 0.5% final formic acid (FA) and the peptides were measured using the Pierce^™^ Quantitative Colorimetric Peptide Assay. The peptides were subjected to simple C18 clean up, and LC-MS/MS analysis was carried out using a nano-LC 1200 system coupled to Orbitrap Lumos ETD mass spectrometer (Thermo Fisher). 1μg peptide was loaded on a two-column setup with precolumn (2 cm × 100 μmI.D.) and analytical column (20 cm × 75 μmI.D.) filled with Reprosil-Pur Basic C18 (1.9 μm, Dr. Maisch GmbH, Germany) as described previously^59^. The MS raw data was searched using Proteome Discoverer 2.1 software (Thermo Fisher) with Mascot algorithm against mouse NCBI refseq database updated 2020_0324. The precursor ion tolerance and product ion tolerance were set to 20 ppm and 0.5Da, respectively. Maximum cleavage of two with Trypsin enzyme, dynamic modification of oxidation (M), protein N-term acetylation and deamidation (N/Q) were allowed. For statistical assessment, missing value imputation was employed through sampling a normal distribution N (μ−1.8 σ, 0.8σ), where μ, σ are the mean and standard deviation of the quantified values. The median normalized and log_10_ transformed iBAQ values (Adj-iBAQ) were used for data analysis. Targets with low Adj-iBAQ values (<100) in the mitochondrial protein from WT and *Ins2*^*Akita/+*^ mice were excluded from the analysis, because these low abundant proteins greatly exaggerated the ratios between *Ins2*^*Akita/+*^ and WT. For complexsome profiling, digitonin-solubilized mitochondria proteins were subjected to BN-GEL analysis. Five putative SC bands, determined as described previously^32^ and in Extended Data Fig. 4f, were excited and cut into 1×1 mm pieces followed by in-gel digestion using LysC and trypsin enzymes. The peptides were dried in a speed vac and dissolved in 10 μl of 5%methanol containing 0.1% FA buffer. LC-MS/MS analysis was conducted in the same way as described above. The peptides identified from mascot result file were validated with 5% false discover rate (FDR). The gene product inference and quantification were done with label-free iBAQ approach using ‘gpGrouper’ algorithm^60^.

### APEX proximity labeling

APEX proximity labeling was performed as previously described^61,62^. In brief, 80–90% confluent cells were pretreated with 500 μM biotin-tyramide for 30 min, followed by 1 mM H_2_O_2_ for 1min. After quenching and washing with PBS containing 5 mM Trolox and 10mM sodium ascorbate, cells were harvested into PBS. The cells were centrifuged, and cell pellets were lysed in RIPA buffer with 1 mM PMSF, 5 mM Trolox, 10 mM sodium ascorbate, and 10 mM sodium azide. Cell lysates were incubated with prewashed Pierce streptavidin magnetic beads (Thermo Fisher) overnight in a cold room. The beads were washed with RIPA buffer (Teknova) twice, with 1 M KCl, 0.1 M Na_2_CO_3_, and 2 M urea in 10 mM Tris-HCl (pH 8.0) once, and with RIPA buffer twice. Bound biotinylated proteins were eluted in 1 x SDS sample buffer and separated on 4–20% PAGE, followed by immunoblotting with NDUFS4 antibody. The immuno-precipitated samples were resolved on NuPAGE 10% Bis-Tris Gel. Each lane was excised into 4 equal pieces and combined into two tubes after in-gel digestion using LysC and trypsin enzymes. Digested peptide was processed and used for the LC-MS/MS analysis in the same way as complexsome profiling.

### Co-immunoprecipitation (Co-IP)

Co-IP of NDUFS4 and STOML2 was carried out as previously described^63^. Briefly, transfected cells were washed and crosslinked with 1 mM dithiobis (succinimidyl propionate) (Sigma) in PBS for 30 min, and quenched with 50 mM Tris (pH 8.0) for 3 min. Cell pellets from scraped suspension were lysed in 0.2ml RIPA buffer (Teknova) on ice for 15 min, diluted with 0.8 ml of NETN buffer (170 mM NaCl, 1 mM EDTA, 50 mM Tris, pH 7.3, 0.5% NP-40) and incubated for another 15 min. Cleared lysate were then incubated with Anti-FLAG M2 Magnetic Beads (Sigma) overnight in cold room. Bound complexes were washed 5 times with NETN buffer and eluted with 100 μg/ml of 3X FLAG peptide (Sigma).

### GST pull-down assay

GST pull-down was carried out as previously described^51^. C-terminus HA-tagged STOML2 wild type and deletion mutants were transiently transfected into HEK 293T cells and then lysed in TNTE buffer (10 mM Tris HCl, pH 7.8, 150 mM NaCl, 1 mM EDTA, and 1.0% Nonidet P-40). Clear lysate was incubated at 4°C for 3hrs with 2 μg GST or GST-NDUFS4 protein immobilized on Glutathione Sepharose 4B beads (GE Healthcare). GST-NDUFS4-bound complex was washed in TNTE buffer five times, separated on SDS-PAGE and analyzed by immunoblotting.

### STED/STORM imaging

STED imaging was performed on an STEDYCON (Abberior) and Eclipse Ti2 inverted imaging system (Nikon Instruments) with a Plan Apochromat 100X (NA 1.49) oil immersion objective (Nikon Instruments). The STEDYCON STED unit was equipped a with 561 nm and 640 nm pulsed excitation lasers and a 775 nm pulsed depletion laser and adjustable pinhole (64 μm) and fluorescence detected by APD detectors after spectral filtering for the orange (650–710 nm) and red channels (550–615 nm), respectively. For imaging of NDUFS4, STAR-Orange was excited at 561 nm and its fluorescence emission was detected at 616 nm with 1–7 ns time gating and STAR-RED excitation used for STOML2 at 640 nm and the emission collected at 640 nm with the same time gating. The two channels were acquired sequentially, using pixel dwell times of 17 μs with a 15 nm pixel size. Mander’s coefficients^64^ were calculated as the indexes of intensity based colocalization, and distance of particle’s center between NDUFS4 and STOML2 were obtained using JACoP plugin of ImageJ^65^. Object-based colocalization was defined as the distance less than 140 nm^66^. For STORM imaging, imaging experiments were conducted with a N-STORM (Nikon Instruments) on an Eclipse Ti2 inverted microscope. STORM images were collected in a 512 ×512-pixel region of interest using a CFI Apochromat TIRF 100x (NA 1.49) oil object (Nikon Instruments) and a C11440–22CU ORCA-flash sCMOS 4.0 V2 camera (Hamamatsu). Images were acquired sequentially 500 frames per filter channel at 200 ms time duration. Cells labeled with Alexa Fluor 647 and Atto 488 secondary antibodies were excited with 100% laser power from a 647 nm and a 488 nm laser, respectively. Nikon Nd2 files were separated and converted to tiff files per channel by custom python script. Single-molecule localization and nearest neighboring distance (NND) were determined by using ThunderSTORM plugin of image J^67^ with default setting except the camera setting: pixel size is 65 nm, photoelectrons per A/D count is 0.57. A set of post processing methods with default setting were applied to optimize the localization data. In each cell, median NND and % colocalization were calculated using a total of data in four non-overlapping representative regions of 20 × 20 μm^2^. Maximum intensity projection (MIP) images were reconstructed from Nikon Nd2 files using Huygens Essential (Scientific Volume Imaging).

### Cryo-ET

Cryo-grids were prepared by plunge-freezing in a liquid ethane using a Vitrobot Mark IV (Thermo Fisher) that was set to 100 % humidity at 4°C. 2 μl of purified mitochondria sample was applied to 200 mesh, R 2/1 Quantifoil copper grids (Quatifoil) and blotted with Whatman filter paper for 4 sec. Cryo-ET data collection was performed using a Titan Krios G3 300 keV FEG transmission electron cryo-microscope (Thermo Fisher). Cryo-ET images were acquired using a BioQuantum energy filter (Gatan) with slit width set to 20 eV. Images were recorded on a 4k × 4k K2 Summit direct electron detector (Gatan) operated in counting mode at nominal microscope magnifications of 26,000x, 33,000x or 19,500x corresponding to pixel sizes of 5.32 Å, 4.20 Å and 7.50 Å for NG, HG and HG-DOX, respectively. SerialEM software^68^ was used for imaging. Each tilt series was collected from −50° to +50° with increment of 2° using the low dose functions for tracking and focusing. The cumulative dose of each tilt-series was 80–90 e^−^/Å^2^. Defocus values were set between −8 μm and −10 μm. For tomogram reconstruction and segmentation, raw movie frames of tilt-series were corrected for beam-induced motion using MotionCor2^69^. The aligned micrographs were imported into EMAN2^70^ and were compiled into tilt-series. Automated alignment was performed and 1k × 1k 3D tomograms (bin4) were generated using e2tomogram.py in EMAN2 software package^70^. The references containing features of interest were manually boxed out in tile images of 64 × 64 pixels using EMAN2 graphical tool and then used for training Convolutional neural network (CNN). Once a CNN has been trained to recognize a certain feature, it was applied to annotate the same feature in a tomogram. Molecular graphics and visualization were performed with UCSF Chimera^71^.

### Protein structure prediction and molecular docking

Human STOML2 structure was predicted by Contact-guided Iterative Threading ASSEmbly Refinement web tool (C-I-TASSER) ^72^. Human NDUFS4 within the structure of the respirasome was obtained from the PDB database (5XTB) ^73^. The ClusPro 2.0 web server^37^ was used to perform protein-protein docking simulation. The resulting docking structures were analyzed using PyMol Molecular Graphics Systems, version 2.0, Schrodinger, LLC.

### Statistics

Group data are expressed as mean ± SEM or median ± IQR. Comparisons between two groups were performed using two-sided unpaired Student’s *t*-test for normally distributed data and two-sided Mann-Whitney test for non-normally distributed data. Comparisons of multiple groups were performed using one way-analysis of variance (one-way ANOVA) followed by Tukey’s multiple comparisons test or Kruskal-Wallis followed by Dunn’s multiple comparisons test based on the sample distribution. Multiple Mann-Whitney test (Fig. 1c) and Student’s *t*-test (Extended Data Fig. 1c–e) were followed by two-stage linear step-up procedure with the specific FDR indicated in the figure legend. All tests were performed with GraphPad version 9.3.1 (Graphpad Software), and *P* values <0.05 were considered to be statistically significant.

## Extended Data

## Supplementary Material

Supplement 1

## Data Availability

All data supporting the findings of this study are available in the main text or supplementary materials. The mass spectrometry proteomic data for mitochondrial proteome in murine podocytes, NDUFS4-APEX proteome, and murine podocyte complexsome have been deposited to the ProteomeXchange Consortium via the PRIDE partner repository with the dataset identifies, PXD041202, PXD041378, and PXD041203, respectively.
