# Supplementary material for: NDUFS4 Regulates Cristae Remodeling in Diabetic Kidney Disease
Source: Res Sq. 2023 Jun 30:rs.3.rs-3070079. Preprint. [Version 1] doi: 10.21203/rs.3.rs-3070079/v1 (PMC10350115; doi:10.21203/rs.3.rs-3070079/v1)
Supplement: Supplement 1 [file NIHPPRS3070079V1-supplement-1.pdf]

Supplementary Table 1. Clinical parameters of donors and DKD patients at renal biopsy.

Clinical parameters	Donor (n=9)	DKD (n=34)	P-value
Male (%)	44	59	0.48
Age (years)	56 ± 13	57 ± 12	0.93
BMI (kg/m^2)	20.9 ± 2.5	25.6 ± 5.4	0.01
Duration of DM (years)*	-	5.5 (2.0-15.0)	
HbA1c (%)	5.7 ± 0.2	7.2 ± 1.6	0.01
SBP (mmHg)	115.8 ± 12.6	146.0 ± 21.7	<0.001
DBP (mmHg)	70.4 ± 12.9	83.1 ± 9.5	<0.01
MAP (mmHg)	85.6 ± 10.5	104.1 ± 11.7	<0.001
Hypertension (%)†	33	85	<0.01
Retinopathy (%)	-	62	
sCr (mg/dl)*	0.71 (0.60-0.84)	1.1 (0.91-1.78)	<0.01
eGFR (ml/min/1.73m^2)	79.0 ± 7.1	51.7 ± 24.7	<0.01
CKD GFR Categories (G1/G2/G3/G4/G5, %)	11/89/0/0/0	6/35/35/18/6	
UACR (mg/gCr)*	6.6 (4.3-13.0)	1590 (629-3030)	<0.001
Normo/Micro/Macro-1/Macro-2 (%)	100/0/0/0	12/6/27/55	

Abbreviations; BMI, body mass index; Duration of DM, estimated duration of diabetes mellitus; SBP, systolic blood pressure; DBP, diastolic blood pressure; MAP, mean arterial pressure; Retinopathy, diabetic retinopathy; sCr, serum creatinine; eGFR, estimated glomerular filtration rate; CKD GFR Categories G1 ≥90 ml/min/1.73m^2, G2 60-90 ml/min/1.73m^2, G3 30-59 ml/min/1.73m^2, G4 15-29 ml/min/1.73m^2, G5 <15 ml/min/1.73m^2; UACR, urinary albumin creatinine ratio; Normo/Micro/Macro: normoalbuminuria, microalbuminuria and macroalbuminuria. Macroalbuminuric patients were divided into: Macro-1: >300 but ≤1000mg/gCr of albuminuria and Macro-2: >1000mg/gCr of albuminuria. *Median (interquartile range). †Hypertension was defined as blood pressure ≥ 140/90 mmHg or the use of antihypertensive drugs.

Supplementary Table 2. List of primer sequences used for qRT-PCR analysis in this study.

Gene	Forward Primer (5'-3')	Reverse Primer (5'-3')
Actb	CTAAGGCCAACCGTGAAAAG	ACCAGAGGCATACAGGGACA
Hprt	AGGGATTTGAATCACGTTTG	TTTACTGGCAACATCAACAG
Nephrin (Nphs1)	CCCAGGTACACAGAGCACAA	CTCACGCTCACAACCTTCAG
Ndufs4	GTCTGTAGAGTTCCATCCAG	GAGCAGGAACAAAGATTCTG
Ndufa2	GAACAATCTGAGTGCTGATG	CGTAAGCTTTATAAGGACCC
Ndufb3	GGTAGCTTTGGGGGCTGAAT	GTCACAAGGCGCTCTCTTCA
Ndufb4	TTTTCAAAACAGACAGGGAC	ATCCTTGCCGAACTTAGTAG
Ndufb5	GTCGTCAAGCCTTCTTTATAC	TCGGATGCTTGTAATACTCC
Ndufb8	CATGTGTAAACATCTCTTCGG	TCCTCAGATATCATAGTGAACC
Ndufb11	CTCAAAAACAACTCTCTCCC	AAGTTTGTTTACGAGTTCGG
Ndufv3	AAAGTGTGCTCAAAGATGTG	TTTCTTGACAAATGCTTCGG

Supplementary Table 3. List of antibodies and dyes used in this study.

**Primary antibodies**	**Source**	**Catalog number**
Anti-GST Alexa Fluor 680	Santa Cruz	sc-138 AF680
Goat anti-Podocalyxin, biotinylated	R&D Systems	BAF1556
Guinea pig anti-Synaptopodin	Progen	GP94-N
Mouse anti-β-Actin	Cell Signaling	4967
Mouse anti–FLAG M2 monoclonal	Sigma-Aldrich	F3165
Mouse anti-STOML2	Proteintech	60052-1-lg
Mouse anti-VDAC	Abcam	ab14734
Mouse OXPHOS cocktail	Thermo Fisher	45-8099
Rabbit anti-ATAD3A/B	Proteintech	16610-1-AP
Rabbit anti-HA-Tag	Cell Signaling	3724
Rabbit anti-Mitofilin	Proteintech	10179-1-AP
Rabbit anti-Ndufs4	Nobus	NBP1-31465
Rabbit anti-Ndufs4	Abcam	ab137064
Rabbit anti-OPA1	BD Biosciences	612606
Rabbit anti-PAX8	Proteintech	10336-1-AP
Rabbit anti-Podocin	Sigma-Aldrich	P0372
Rabbit anti-STOML2	Proteintech	10348-1-AP
Rabbit anti-VDAC	Cell Signaling	4661
Rabbit anti-Wilms Tumor Protein	Abcam	ab89901
Sheep anti-KIRREL3, biotinylated	R&D Systems	BAF4910

**Secondary antibodies**	**Source**	**Catalog number**
Donkey anti–mouse Alexa Fluor 488	Thermo Fisher	A21202
Donkey anti–mouse Alexa Fluor 594	Thermo Fisher	A21203
Donkey anti–rabbit Alexa Fluor 488	Thermo Fisher	A21206
Donkey anti–rabbit Alexa Fluor 594	Thermo Fisher	A21207
Donkey anti–rabbit Alexa Fluor 647	Thermo Fisher	A21244
Goat anti-guinea pig Alexa Fluor 594	Thermo Fisher	A11076
Goat anti–mouse DyLight 680	Thermo Fisher	35519
Goat anti–mouse DyLight 800	Thermo Fisher	SA510172
Goat anti-mouse ATTO 488	Rockland	610-152-121
Goat anti–rabbit DyLight 680	Thermo Fisher	35568
Goat anti–rabbit DyLight 800	Thermo Fisher	SA535571
Goat anti–rabbit IgG HRP Polymer	Vector Laboratories	MP-7451

**Dyes**	**Source**	**Catalog number**
DAPI	Thermo Fisher	62248
Mitotracker Red	Thermo Fisher	M7512

Supplementary Files

This is a list of supplementary files associated with this preprint. Click to download.

- Reportingsummarycopy.pdf
- NCOMMS2327258TRS.pdf
